# Bioinformatics Prediction and Evolution Analysis of Arabinogalactan Proteins in the Plant Kingdom

**DOI:** 10.3389/fpls.2017.00066

**Published:** 2017-01-26

**Authors:** Yuling Ma, Chenchao Yan, Huimin Li, Wentao Wu, Yaxue Liu, Yuqian Wang, Qin Chen, Haoli Ma

**Affiliations:** ^1^State Key Laboratory of Crop Stress Biology for Arid Areas, College of Agronomy, Northwest A&F UniversityYangling, China; ^2^National Base for the Talents on Life-Science and Technology, Innovation Experimental College, Northwest A&F UniversityYangling, China

**Keywords:** arabinogalactan proteins, bioinformatics, chimeric AGP, evolution, Finding-AGP program

## Abstract

Arabinogalactan proteins (AGPs) are a family of extracellular glycoproteins implicated in plant growth and development. With a rapid growth in the number of genomes sequenced in many plant species, the family members of AGPs can now be predicted to facilitate functional investigation. Building upon previous advances in identifying *Arabidopsis* AGPs, an integrated strategy of systematical AGP screening for “classical” and “chimeric” family members is proposed in this study. A Python script named Finding-AGP is compiled to find AGP-like sequences and filter AGP candidates under the given thresholds. The primary screening of classical AGPs, Lys-rich classical AGPs, AGP-extensin hybrids, and non-classical AGPs was performed using the existence of signal peptides as a necessary requirement, and BLAST searches were conducted mainly for fasciclin-like, phytocyanin-like and xylogen-like AGPs. Then glycomodule index and partial PAST (Pro, Ala, Ser, and Thr) percentage are adopted to identify AGP candidates. The integrated strategy successfully discovered AGP gene families in 47 plant species and the main results are summarized as follows: (i) AGPs are abundant in angiosperms and many “ancient” AGPs with Ser-Pro repeats are found in *Chlamydomonas reinhardtii*; (ii) Classical AGPs, AG-peptides, and Lys-rich classical AGPs first emerged in *Physcomitrella patens, Selaginella moellendorffii*, and *Picea abies*, respectively; (iii) Nine subfamilies of chimeric AGPs are introduced as newly identified chimeric subfamilies similar to fasciclin-like, phytocyanin-like, and xylogen-like AGPs; (iv) The length and amino acid composition of Lys-rich domains are largely variable, indicating an insertion/deletion model during evolution. Our findings provide not only a powerful means to identify AGP gene families but also probable explanations of AGPs in maintaining the plant cell wall and transducing extracellular signals into the cytoplasm.

## Introduction

Arabinogalactan proteins (AGPs) are a subfamily of hydroxyproline-rich glycoproteins (HRGPs) and implicated in many processes of plant growth and development (Seifert and Roberts, 2007; Kavi Kishor et al., 2015). AGPs consist of a protein backbone and carbohydrate side chains rich in arabinose and galactose (Ellis et al., 2010; Showalter and Basu, 2016). In most conditions, glycosylphosphatidylinositol (GPI) anchor signals are present in the C-terminal of AGPs (Borner et al., 2003). AGPs are highly glycosylated, namely the percentage of carbohydrate is often more than 90% and the molecular mass is above 60–300 kD (Seifert and Roberts, 2007; Ellis et al., 2010; Hijazi et al., 2014a; Nguema-Ona et al., 2014). AGPs are glycoproteins with high heterogeneity due to the arrangement patterns and variable contents of different monosaccharaides (Gaspar et al., 2001).

Based on variable core protein backbones, AGPs were generally classified as classical AGPs and non-classical AGPs (Showalter, 1993, 2001). Protein backbones of classical AGPs usually consisted of three parts: an N-terminal signal peptide; single central domain with varying length and rich in Pro, Ala, Ser, and Thr (PAST) residues; and a C-terminal GPI anchor signal (Schultz et al., 2000). The PAST-rich domains of one type of classical AGPs are usually separated by Lys-rich regions and termed as Lys-rich classical AGPs (Li and Showalter, 1996; Gilson et al., 2001; Sun et al., 2005). The other kind of classical AGPs, termed as AG peptides because of its mature protein backbone, are only 10–15 amino acids in length. There are also many chimeric AGPs with different conservative domains that could be classified into three main subfamilies: fasciclin-like AGPs (FLA; Johnson et al., 2003; Ma and Zhao, 2010; MacMillan et al., 2015), phytocyanin-like AGPs (PAG; Mashiguchi et al., 2009; Ma et al., 2011), and xylogen-like AGPs (XYLP; Motose et al., 2004; Kobayashi et al., 2011). In addition, AGPs with sequence characteristics of both AGPs and extensins (EXT) are termed as AGP-extensin hybrids (HAE; Showalter et al., 2010).

Typical AGPs are rich in PAST and these amino acids are regularly arranged as Ala-Pro, Ser-Pro, and Thr-Pro, which were introduced as arabinogalactan (AG) glycomodules (Shpak et al., 1999; Ellis et al., 2010). Previous studies have used synthetic peptides to examine glycosylation patterns that refer to special arrangements. Pro that is non-contiguously present in repeated sequences of Ala-Pro and Ser-Pro are totally hydroxylated for glycosylation and the glycans are rich in arabinose and galactose. Meanwhile, Pro contiguously arranged in Ser-Pro_2−4_ are also hydroxylated and the main component of the glycan is arabinose except that both arabinosides and arabinogalactan polysaccharides were found in the carbohydrate of Ser-Pro_3_ repeats (Shpak et al., 1999, 2001). These experiments led to the Hyp-contiguity hypothesis, that states contiguous Hyp (e.g., Ser-Hyp_2−4_) are mainly glycosylated by arabinoses, while non-contiguous Hyp are glycosylated by AG. Several studies have proved this hypothesis by using a specific reagent called β-glucosyl Yariv (β-GlcY) that could bind with the carbohydrate moieties of AGPs, which was used to purify AGPs and further examine the glycosyl composition and the distribution patterns of Hyp. On the basis of this method, studies have already proved that at least 19 proteins in *Arabidopsis* are glycosylated by AG, including classical AGPs, FLA, PAG, and AG-peptides (Schultz et al., 2000; Johnson et al., 2003; Hijazi et al., 2012). There are also several β-GlcY reactive AGPs in *Oryza sativa* namely OsAGP1, OsAGPEP1, OsAGPEP2, OsAGPEP3, OsENDOL1, and OsLTPL1 (Mashiguchi et al., 2004). Although X-Pro repeats (where X represents Ala, Ser, or Thr) are present in a lot of known AGPs, there are also some exceptions without non-contiguous X-Pro repeats. For example, AG modified SOS5/FLA4 (Salt Overly Sensitive 5/Fasciclin-like AGP 4) only contains TPPPT and SPPPA motifs, and three PPAKAPIKLP repeats are found in AtAGP30 (Shi et al., 2003; van Hengel and Roberts, 2003; Griffiths et al., 2016). By analyzing mutated sequences of sporamin, it was found that Pro located in amino acid sequences, such as [not basic]-[not T]-[AVSG]-Pro-[AVST]-[GAVPSTC]-[APS], are efficiently AG glycosylated (Shimizu et al., 2005).

On the basis of biased amino acid compositions and special sequence arrangements, recent approaches use bioinformatics to identify AGPs from *Arabidopsis* and rice (Schultz et al., 2002; Ma and Zhao, 2010; Showalter et al., 2010). An excellent Perl script called “amino acid bias” can effectively distinguish PAST-rich proteins from others with certain thresholds (e.g., >50%, Schultz et al., 2002). However, chimeric AGPs with a relatively low PAST proportion are not easily discovered by using amino acid bias. A series of studies identified chimeric AGPs by homology searching of FLA, XYLP, and PAG across genome databases of *Arabidopsis*, rice, wheat, cabbage, eucalyptus, and poplar (Johnson et al., 2003; Faik et al., 2006; Mashiguchi et al., 2009; Ma and Zhao, 2010; Kobayashi et al., 2011; Ma et al., 2011, 2014; Li et al., 2013; MacMillan et al., 2015; Zang et al., 2015). Furthermore, the BIO OHIO software is developed to identify and classify AGPs, EXTs, proline-rich proteins (PRPs), and hybrid HRGPs in *Arabidopsis*. Typically, Ala-Pro, Pro-Ala, Ser-Pro, and Thr-Pro counts are used to evaluate AGPs in addition to calculating the proportion of PAST (Showalter et al., 2010). Recently, the newly released version 2.0 of BIO OHIO was used to identify the HRGPs of *Populus trichocarpa*, including 162 AGPs, 60 EXTs, and 49 PRPs (Showalter et al., 2016). Although whole genome sequences of many plant species have been released, to date only the entire gene families of *Arabidopsis* and rice are systematically analyzed. Building upon previous studies referred to amino acid bias and BIO OHIO, we develop a program named “Finding-AGP” to identify entire AGP gene family from mass data. Compared with previous advances in identifying AGPs, the Finding-AGP program could not only identify AGPs with high PAST percentage (>50%) but also cover most chimeric AGPs with low PAST percentage. Because the main processes of post translational modifications including Pro hydroxylation and AG glycosylation were happened in the endomembrane system including endoplasmic reticulum and Golgi apparatus (Gaspar et al., 2001; Nguema-Ona et al., 2014), and most predicted AGPs and all confirmed AGPs by monosaccharide composition analysis were predicted to be secreted (Schultz et al., 2000; Johnson et al., 2003; Mashiguchi et al., 2004; Hijazi et al., 2012), the presence of N-terminal signal peptide was used a dichotomous variable to reduce the number of false positives. The AG glycomodules were determined by statistical analyses of the amino acid compositions of 87 representative AGP-like sequences. The motif of successful AG glycosylation was defined to be at least three glycomodules which were interspaced by no more than 10 amino acid residues. Based on above descriptions, seven variables were incorporated into the Finding-AGP program to find AGP-like sequences, including total length, total PAST percentage, total glycomodule number, partial length, partial PAST percentage, partial glycomodule number, and glycomodule index. Moreover, we used the Finding-AGP program to identify the entire AGP gene families of 47 selected plant species. The most important contribution of this study is in finding a more accurate and effective way to identify AGPs.

## Materials and methods

### Development and basic operations of the finding-AGP script

A Python script named Finding-AGP was written on PyCharm Edition 5.0.3 to find AGP-like sequences and calculate the sequence characteristics of whole protein sequences and AGP-like sequences (part of whole protein sequences), which could be used on Microsoft Windows and Linux CentOS systems. In this study, the glycomodules were determined to be Ala-Pro, Pro-Ala, Ser-Pro, Pro-Ser, Thr-Pro, and Pro-Thr, and there were at least three glycomodules in corresponding AGP-like sequence. The Finding-AGP script could screen for AGP candidates using seven variables under user-defined parameters, including the length of whole protein sequence (Length_T_) and AGP-like sequence (Length_P_), the PAST percentage in whole protein sequence (PAST_T_%) and AGP-like sequence (PAST_P_%), the glycomodule number of the whole protein sequence (GlycoNo_T_) and AGP-like sequence (GlycoNo_P_), and the glycomodule index of the AGP-like sequence (GlycoIndex). The input files were compatible with multiple formats, such as pep, fasta, and txt. The output files contained two txt files. One txt file included the protein identifiers meeting the criteria, values of the seven variables, and the AGP-like sequences of the corresponding identifier. The other output was the sequences of AGP candidates in fasta format.

### Publicly available data collection

A wide range of sequenced plant species were used in the present study, including 33 species of eudicot plants and 10 species of monocot plants. There were also one species each in gymnosperm, pteridophyte, bryophyta, and chlorophyta. The annotated protein sequences of most species were downloaded from Phytozome V11 (https://phytozome.jgi.doe.gov/pz/portal.html) and the others were obtained from genome sequencing databases of the species (database websites and data versions were listed in Supplementary Table 5).

### Signal peptide predictions

The portable version of SignalP 4.1 available for Linux was requested from the SignalP website (http://www.cbs.dtu.dk/services/SignalP/) (Petersen et al., 2011) and installed on a computer with a Linux CentOS system. The Perl (Version 5.6 or higher) and GNUPLOT (Version 4.0 or higher) programs must be already installed for successful running of SignalP 4.1. The number of input sequences allowed per run (MAX_ALLOWED_ENTRIES) was edited in the top of the file “signalp” and the value was set as 100,000 which were more than the greatest number of proteins in all selected species. The sensitive mode was used to judge whether there was a signal peptide in the N-terminal of a protein, namely *D*-value cutoff was more than 0.34.

### BLASTP searches

Local BLAST analyses were performed using the stand-alone BLAST application (version ncbi-blast-2.2.28+). Text-based commands were input to run utilities through a command window. The protein sequences of 47 species in fasta format were reformatted into database files suitable for the BLAST application using the command of formatting database. Then, the protein sequences of known AGPs in fasta format were used as seed sequences to obtain homologous proteins using BLASTP utility with a cutoff *e*-value (*e*^−3^).

### Key bioinformatics websites and settings

A series of bioinformatics websites were used in this study. (i) The phylogenetic relationship of 47 selected plant species was determined by the common tree taxonomy tool at NCBI (http://www.ncbi.nlm.nih.gov/Taxonomy/CommonTree/wwwcmt.cgi) and referred to the phylogenetic tree of the species in Phytozome (https://phytozome.jgi.doe.gov/pz/portal.html). (ii) The signal peptides were also predicted on SignalP 4.1 Server (http://www.cbs.dtu.dk/services/SignalP/) in addition to running on local laptop, the input files were in fasta format and the *D*-cutoff values (0.34) were set in the sensitive mode. (iii) The GPI-anchored signals were determined on big-PI Predictor-GPI Modification Site Prediction (http://mendel.imp.ac.at/sat/gpi/gpi_server.html). (iv) The first 20 amino acids and last 20 amino acids were excluded before predicting the transmembrane domains, because the N-terminal signal peptides and C-terminal GPI anchor signals were usually predicted to be transmembrane domains. The transmembrane domains of putative AGPs were predicted on TMHMM Server v. 2.0. (v) The conserved domain of chimeric AGPs was determined on the NCBI Batch Web CD-Search Tool (http://www.ncbi.nlm.nih.gov/Structure/bwrpsb/bwrpsb.cgi).

### Searching criteria for AGPs

Based on the definition of AG glycomodules and the previous description of AGPs, AGPs were termed as proteins that contained predominantly glycomodules (at least three) throughout all or partial sequence (except N-terminal secreted, C-terminal GPI-anchored signal, and other conserved regions) of the protein backbones without having repeated sequence corresponding to EXTs or PRPs (e.g., Ser-Pro_2−4_ or PVKCYT; Schultz et al., 2002; Showalter et al., 2010). The classifications of AGP subfamilies were listed as follows: (i) classical AGPs consisted of a N-terminal signal peptide, a PAST-rich region of variable length, and a GPI-anchored signal; (ii) AG-peptides were a subclass of classical AGPs of short length (<90 amino acid residues); (iii) Lys-rich classical AGPs were another subclass of classical AGPs, their PAST regions were spaced by a short Lys-rich region; (iv) AGPs were classified as chimeric if there were atypical regions in addition to the PAST-rich region except N- and C-terminal signals; (v) There were also a subclass of AGPs named AGP-extensin hybrids that have characteristics of AGPs and extensins.

### Multiple sequence alignment and phylogenic analysis

The full-length amino acid sequences of Lys-rich classical AGPs were used in multiple sequence alignments, which were performed using ClustalX (version 1.83) with default settings. An unrooted phylogenetic tree was generated using MEGA 6.0 with the neighbor-joining method and bootstrapping was performed 1000 times.

## Results

### Statistical analyses on amino acid compositions of AGP-like sequences

According to the biased amino acid composition (high PAST percentage, usually >50%) and specific arrangement of Ala-Pro, Pro-Ala, Ser-Pro, and Thr-Pro, AGPs were distinguishable from other kinds of proteins and other subfamilies of HRGP such as EXT and PRP (Schultz et al., 2002; Showalter et al., 2010). In order to reveal the amino acid code of arabinogalactan (AG) glycosylation, we conducted statistical analyses on amino acid compositions of 325 known AGPs from 22 plant species. The 325 AGPs included 42 classical AGPs, 9 Lys-rich AGPs, 40 AG-peptides, 5 HAE, 98 FLA, 74 PAG, 35 XYLP, and 22 non-classical AGPs (Supplementary Tables 1, 2). First, we calculated the total number of each amino acid (20 in total) and the frequency of given amino acids, then the percentage of each amino acid in all amino acid residues and the average number per sequence were also determined (Supplementary Table 3). Seven types of amino acids including Ala (13.09%), Ser (10.76%), Pro (10.47%), Leu (8.55%), Val (7.51%), Thr (7.10%), and Gly (6.75%) accounted for almost half of all amino acids (50.38%) and were present in all 325 sequences except for Thr which was absent in PpAGP5. Obviously, the amino acid compositions of signal peptides, GPI-anchor signals, and conserved domains (e.g., fasciclin-like, etc.) significantly affected correct estimation of AGP-like sequences. Thus, a total of 87 classical AGPs, Lys-rich classical AGPs, and AG-peptides (short classical) were selected to represent the characteristics of AGP-like sequences. Meanwhile, the signal peptides and GPI-anchor signals were removed at most possible cleavage sites to avoid interference caused by these domains. The statistical analyses presented in Supplementary Table 3 were also conducted on AGP-like sequences of the 87 AGPs mentioned above (Table 1). As a result, we found that 1772 residues of Pro accounted for 27.58% of all amino acids and were the most abundant of the 20 amino acids, indicating that these sequences were family members of HRGPs. The number of Ala, Ser, and Thr were 1313, 899, and 566 in total, respectively, and accounted for 20.44, 13.99, and 8.66% of all amino acids, respectively. It was noteworthy that Pro and Ala were presented in all 87 selected sequences. Compared with amino acid compositions of 325 known AGPs in full-length, the order of the seven most abundant amino acids changed from “Ala, Ser, Pro, Leu, Val, Thr, Gly” to “Pro, Ala, Ser, Thr, Val, Gly” in the analyses of 87 AGP-like sequences, indicating that the enrichment of Leu was not a leading feature of AGP-like sequences (Table 1 and Supplementary Table 3). It was believed that the high levels of PAST percentage in 87 processed sequences (70.67% of all amino acids) could be used to identify AGP-like sequences.

**Table 1:** **Compositions of 20 amino acids in 87 AGP-like sequences**.

**Amino acid^a^(three letter)**	**Amino acid (one letter)**	**Total number^b^**	**Frequency of given amino acid in 87 sequences**	**Average number of given amino acid per sequence^c^**	**Percentage of given amino acid in all amino acids (%)^d^**
Pro	P	1772	87	20.37	27.58
Ala	A	1313	87	15.09	20.44
Ser	S	899	82	10.96	13.99
Thr	T	556	74	7.51	8.66
Val	V	323	66	4.89	5.03
Gly	G	261	69	3.78	4.06
Glu	E	214	62	3.45	3.33
Lys	K	204	42	4.86	3.18
Asp	D	189	64	2.95	2.94
Leu	L	146	45	3.24	2.27
Gln	Q	114	61	1.87	1.77
Met	M	85	24	3.54	1.32
His	H	84	24	3.5	1.31
Ile	I	65	33	1.97	1.01
Asn	N	62	35	1.77	0.97
Arg	R	48	22	2.18	0.75
Tyr	Y	39	18	2.17	0.61
Phe	F	31	21	1.48	0.48
Cys	C	12	4	3	0.19
Trp	W	7	4	1.75	0.11

a *The order of 20 amino acids was displayed according to their total number from high to low*.

b *The sum of given amino acid in 87 processed sequences*.

c *The value was calculated by using the total number of given amino acid divide the number of sequences with given amino acid*.

d *The value was calculated by using total number of given amino acid divide total number of amino acids in 87 processed sequences*.

Moreover, the notion of glycomodules was used to characterize AGP-like sequences, namely dipeptides like Ala-Pro, Ser-Pro, and Thr-Pro were present in many AGPs. In consideration of this, we analyzed distribution patterns of Pro and other amino acid residues in 87 AGP-like sequences. In other words, we counted the number of X-Pro (X represented any other amino acid except for Pro) in these sequences. The glycomodules like Ala-Pro, Ser-Pro, and Thr-Pro were much more abundant than the other 16 composition modes (X-Pro, X represented any other amino acid except for Pro, Ala, Ser, and Thr) and accounted for 81.06% of non-contiguous Pro residues (Table 2). For the other 16 composition modes, we further counted the number of Ala, Ser, and Thr after Pro (e.g., Gly-Pro-Ala, Gly-Pro-Ser, Gly-Pro-Thr; Supplementary Table 4). The number of Pro-Ala, Pro-Ser, and Pro-Thr was 126 in total and accounted for 9.14% of non-contiguous Pro residues. The specific arrangements of Pro, Ala, Ser, and Thr successfully characterized AGP-like sequences, namely the glycomodules Ala-Pro, Pro-Ala, Ser-Pro, Pro-Ser, Thr-Pro, and Pro-Thr represented 90.20% of non-contiguous Pro residues in 87 AGP-like sequences. Thus, the method of glycomodule counts could be another important indicator to identify AGP-like sequences.

**Table 2:** **Glycomodule counts in 87 AGP-like sequences**.

**Glycomodule^a^**	**Total number^b^**	**Frequency of given glycomodule in 87 sequences**	**Average number of given glycomodule per sequence^c^**	**Percentage of given glycomodule in all amino acids (%)^d^**
AP	547	87	6.29	39.70
SP	383	61	6.28	27.79
TP	187	44	4.25	13.57
GP	73	40	1.83	5.30
VP	43	27	1.59	3.12
LP	30	18	1.67	2.18
EP	20	17	1.18	1.45
KP	19	12	1.58	1.38
MP	15	4	3.75	1.09
IP	12	10	1.20	0.87
QP	12	9	1.33	0.87
NP	9	9	1.00	0.65
DP	7	5	1.40	0.51
YP	6	5	1.20	0.44
RP	5	3	1.67	0.36
FP	4	4	1.00	0.29
WP	3	1	3.00	0.22
CP	3	1	3.00	0.22
HP	0	0	0.00	0.00

a *The order of 19 putative glycomodule was displayed according to their total number from high to low*.

b *The sum of given glycomodule in 87 processed sequences*.

c *The value was calculated by using the total number of given glycomodule divide the number of sequences with given glycomodule*.

d *The value was calculated by using total number of given glycomodule divide total number of all putative glycomodules in 87 processed sequences*.

### The variables of glycomodule index and partial PAST percentage in identifying AGPs

The definition of AGPs is that glycomodules such as Ala-Pro, Ser-Pro, and Thr-Pro are distributed throughout the sequence and non-contiguous Pro is interspaced by no more than 11 amino acid residues (Schultz et al., 2002). The major differences between classical AGPs, AG-peptides, and chimeric AGPs are the length of AGP-like sequences and the number of glycomodules. Similar to the definition of AGPs in previous studies, we defined that the sequence with glycomodules spaced by no more than 10 amino acid residues might be effectively glycosylated by AG. In our opinion, the criterion that protein sequences with only two glycomodules was less likely to discriminate AGPs and other proteins. For instance, there were more than half of *Arabidopsis* proteins (i.e., 15,851 of 27,416) and nearly two-thirds of rice proteins (i.e., 26,618 of 39,049) have at least two glycomodules. Also, because the shortest AGP-like sequences found in AG-peptides usually had three glycomodules, we defined that the AGP-like sequences consisted of at least three glycomodules. A notion designated as the glycomodule index (GlycoIndex) was proposed in this study to represent the enrichment of glycomodules in AGP-like sequences with variable length. The GlycoIndex could be calculated as the ratio of the number of glycomodules to the length of AGP-like sequence, and the beginning and end of AGP-like sequences were both glycomodules. For example, the AGP-like sequence of AtAGP1 is “SPAPAPSNVGGRRISPAPSPKKMTAPAPAPEVSPSPSPAAALTPESSASPPSPPLADSPTADSPALSPSAISDSPTEAPGPA,” therefore, the GlycoIndex is 0.26 (21/82, glycomodule number/sequence length). If there were two or more AGP-like sequences in one protein, these AGP-like sequences were joined together into one sequence which was then regarded as the representative AGP-like sequence of the corresponding protein.

Moreover, a total of 325 known AGPs were collected to illustrate the method of identifying AGPs. A Python script named “Finding-AGP” was written to extract AGP-like sequences (i.e., at least three glycomodules interspaced by no more than ten amino acid residues). We found that 15 of the 325 known AGPs were not in accordance with our definition of AGPs that contained at least three glycomodules interspaced by no more than ten amino acid residues, including one non-classical AGP, two AG-peptides, three FLA, four PAG, and five XYLP. Consequently, the statistical analyses of a total of 310 known AGPs were conducted to find an effective strategy and searching criteria for identifying AGPs (Supplementary Table 5). Generally, the seven variables, total length (Length_T_), total PAST percentage (PAST_T_%), total glycomodule number (GlycoNo_T_) in whole protein sequences, and partial length (Length_P_), partial PAST percentage (PAST_P_%), partial glycomodule number (GlycoNo_P_), and GlycoIndex in AGP-like sequences, were incorporated in the Finding-AGP program (Table 3, see Section Materials and Methods for details). A correlation analysis was performed to reveal the internal relationships among these seven variables and the correlation coefficients were calculated to the degree of correlation in pairs (Table 4). The regression coefficient analysis showed that very strong correlations (|*r*| > 0.8) were found between GlycoNo_T_ and GlycoNo_P_ (*r* = 0.962), between GlycoNo_P_ and Length_P_ (*r* = 0.961), and between GlycoNo_T_and Length_P_ (*r* = 0.935), indicating that most glycomodules were located in AGP-like sequences and the increase of glycomodules was positively correlated with the length of AGP-like sequences. Furthermore, the efficiencies of variables Length_P_ and GlycoNo_P_ in identifying AGPs were mostly the same as the GlycoNo_T_ that was formerly proposed as a glycomodule count by Showalter et al. (2010). Interestingly, degrees of correlation were low between GlycoIndex and all other variables (|*r*| < 0.5) except PAST_P_% (*r* = 0.724), indicating that the variables GlycoIndex and PAST_P_% could identify AGPs which were not covered by the variables Length_T_ and PAST_T_%. In other words, a large number of AGPs (especially chimeric AGPs) with a high GlycoIndex in the AGP-like sequences could be effective in identifying AGPs with low PAST_T_%. Meanwhile, the PAST_P_% of AGP-like sequences was higher than PAST_T_% because the enrichment of glycomodules in AGP-like sequence consequentially led to an increase of PAST percentage.

**Table 3:** **Seven variables used in identifying AGPs**.

**Variable name**	**Abbreviations**	**Method of calculation**
Total length	Length_T_	The length of whole protein sequence
Total PAST percentage	PAST_T_%	The percentage of Pro, Ala, Ser, and Thr in whole protein sequence
Total glycomodule number	GlycoNo_T_	The number of Ala-Pro, Pro-Ala, Ser-Pro, Pro-Ser, Thr-Pro, and Pro-Thr in whole protein sequence
Partial Length	Length_P_	The length of AGP-like sequence
Partial PAST percentage	PAST_P_%	The percentage of Pro, Ala, Ser, and Thr in AGP-like sequence
Partial glycomodule number	GlycoNo_P_	The number of Ala-Pro, Pro-Ala, Ser-Pro, Pro-Ser, Thr-Pro, and Pro-Thr in AGP-like sequence
Glycomodule index	GlycoIndex	The ratio of GlycoNo_P_ and Length_P_

**Table 4:** **Correlation analysis of seven variables in 310 known AGPs with at least three glycomodules**.

	**Length_T_**	**PAST_T_%**	**GlycoNo_T_**	**Length_P_**	**PAST_P_%**	**GlycoNo_P_**	**GlycoIndex**
Length_T_	1						
PAST_T_%	−0.33^**^	1					
GlycoNo_T_	0.47^**^	0.49^**^	1				
Length_P_	0.29^**^	0.58^**^	0.93^**^	1			
PAST_P_%	−0.29^**^	0.16^**^	−0.13	−0.20^**^	1		
GlycoNo_P_	0.27^**^	0.59^**^	0.96^**^	0.96^**^	−0.07	1	
GlycoIndex	−0.15	−0.16^**^	−0.21^**^	−0.36^**^	0.72^**^	−0.20^**^	1

** *Correlation is significant at the 0.01 level (2-tailed)*.

**Figure 1:**
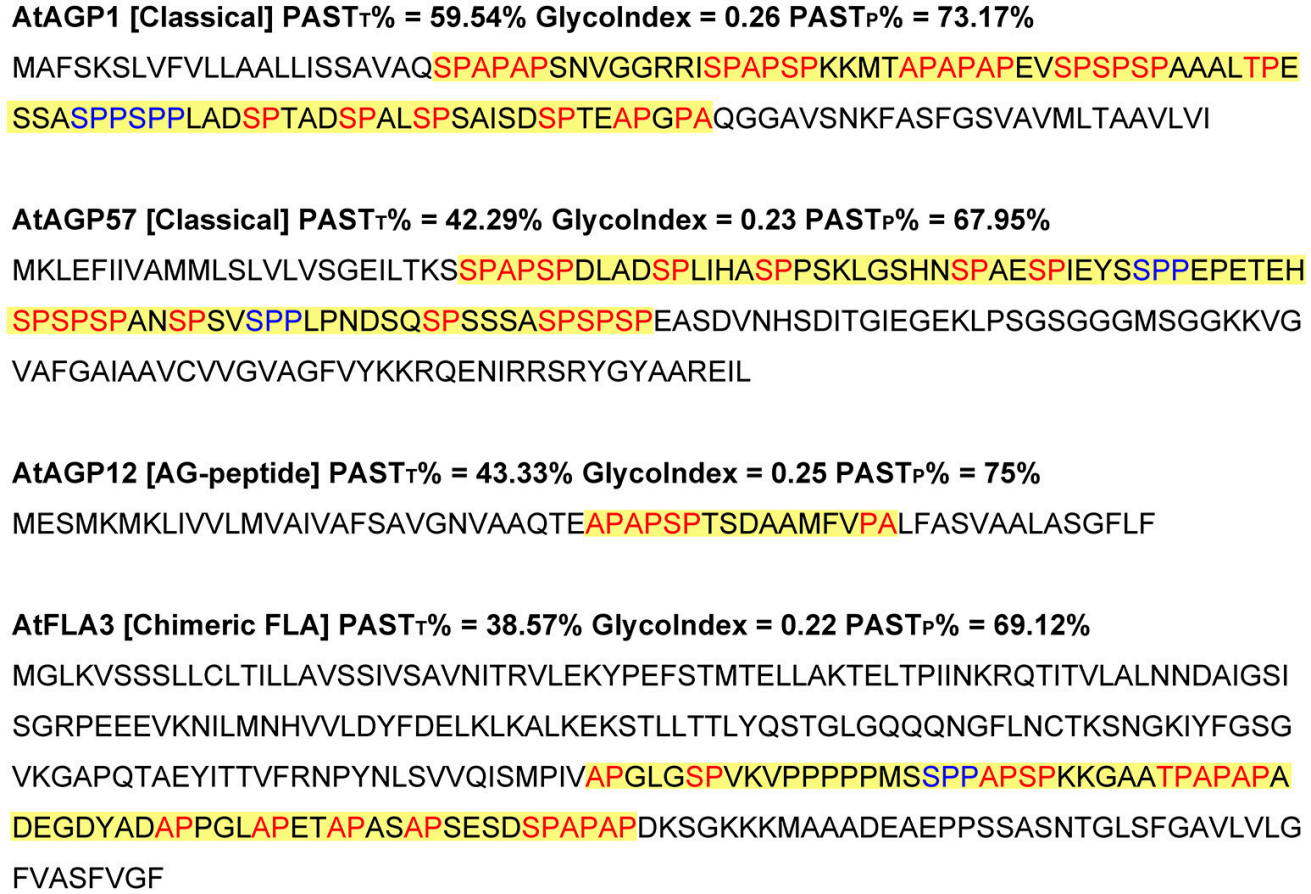
**Illustration of the variables glycomodule index and partial PAST percentage of representative ***Arabidopsis*** AGPs**. Red colored sequences indicate putative AG glycomodules (Ala-Pro, Pro-Ala, Ser-Pro, Pro-Ser, Thr-Pro, and Pro-Thr) and blue colored sequences indicate putative EXT glycomodules (Ser-Pro_2−4_). Total PAST percentage (PAST_T_%) and partial PAST percentage (PAST_P_%) represent the PAST percentage in whole protein sequences and AGP-like sequences, respectively. GlycoIndex, glycomodule index.

Typically, four AGPs from *Arabidopsis* were selected to demonstrate the applicability of the GlycoIndex feature (Figure 1), including AtAGP1 (classical), AtAGP57 (classical), AtAGP12 (AG-peptide), and AtFLA3 (chimeric FLA). The AGP-like sequences of these four AGPs were first obtained and then the PAST_T_%, GlycoIndex, and PAST_P_% were also calculated. The frequently used threshold of identifying AGPs is more than 50% PAST_T_ which could only screen out AtAGP1 (59.54%). If the PAST_T_% was reduced to 35% and at the same time the Length_T_ was below 90, AG-peptides like AtAGP12 were easily identified. However, for AGPs with relatively low PAST_T_% and long Length_T_ (e.g., AtAGP57 and AtFLA3), the PAST_T_% calculation method lost its usability. The GlycoIndex parameter seemed to be a universal feature of AGP-like sequences because these four selected AGPs had relatively high values from 0.22 to 0.26 and the only difference was the variable lengths of their AGP-like sequences. Meanwhile, the PAST_P_% of the AGP-like sequences uniformly arrived at high levels from 67.95 to 75%. Undoubtedly, the high levels of GlycoIndex and PAST_P_% could effectively screen out AGPs with low PAST_T_% (from 38.75 to 43.33%) even if these proteins were variable in Length_T_ and belonging to different subfamilies. Therefore, the variables GlycoIndex and PAST_P_% were used to screen for AGP candidates in this study.

### Strategy and criteria of identifying AGPs

**Figure 2:**
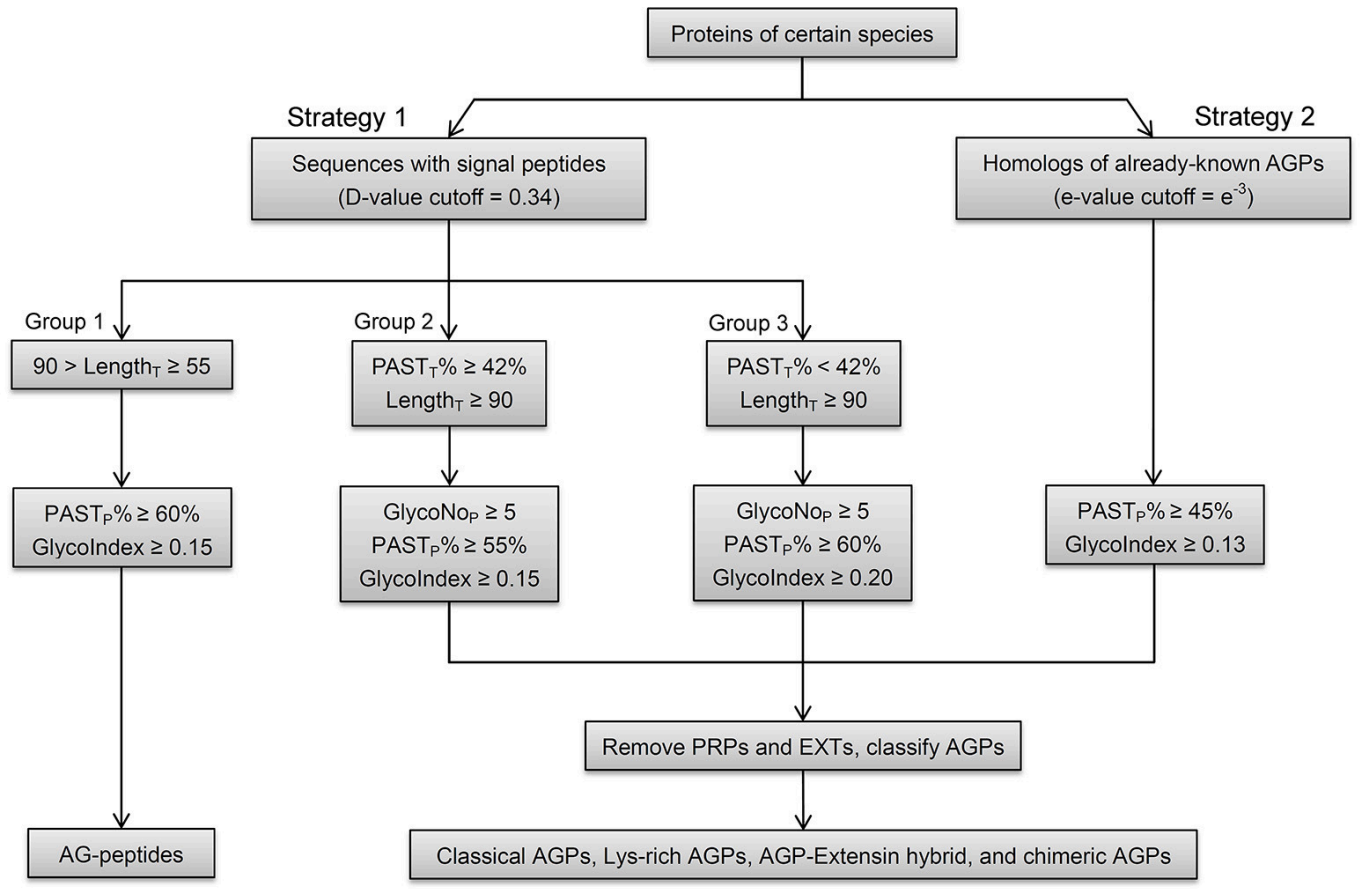
**Schematic workflow of the integrated strategy in identifying AGPs**. Strategy 1 (signal peptide filtration), which includes three groups, is mainly for the identifications of AGPs with relatively high PAST_T_% in given length ranges. The operation of Group 1 identifies AG-peptides. Group 2 and 3 can identify classical AGPs, Lys-rich classical AGPs, AGP-extensin hybrids, and non-classical AGPs. Strategy 2 (BLAST searches) is mainly for the identifications of chimeric AGPs (FLAs, PAGs, and XYLPs) and any other AGPs with high homology to known AGPs. The PAST_P_% and GlycoIndex are used to get PAST-rich AGP-like sequences with glycomodules throughout. The GlycoNo_P_ is also defined as five for both Group 2 and 3. The other types of HRGPs (mainly EXTs and PRPs) are removed and the remaining AGP candidates are classified into classical AGPs, Lys-rich classical AGPs, AGP-extensin hybrid, AG-peptides, chimeric AGPs (FLA, PAG, and XYLP), and other chimeric AGPs (non-classical AGPs are deemed as members belong to other types of chimeric AGPs).

Based on variable length and the presence of signal peptide and conserved domains of AGPs, an integrated strategy was proposed to identify whole gene families (Figure 2). First, because the overwhelming majority of AGPs (295 of 310, 95.16%) were predicted to be secreted to the endoplasmic reticulum for post-translational modification by SignalP4.1 when the cutoff value was set to sensitive mode, a dichotomic variable concerning the existence of signal peptides (i.e., whether there was a signal peptide or not) was proposed to be a necessary requirement of AGP prediction in Strategy 1. To identify as many AGP candidates as possible, especially for AGPs without signal peptides, homologous proteins (Strategy 2) of known AGPs were obtained by using the protein utility of the Basic Local Alignment Search Tool (BLASTP; cutoff value = *e*^−3^). Then, we removed the BLAST search hits from the results of signal peptide prediction and only retained sequences that were not homologous to the known AGPs in Strategy 1.

The BLASTP search could identify all subfamily members belong to FLA, PAG, XYLP, and other AGP candidates with high homology to known AGPs, the signal peptide filtration mainly identified AGPs with low homology, including classical AGPs (C), Lys-rich classical AGPs (KC), AGP-extensin hybrids (HAE), AG-peptide (Pep), and non-classical AGPs (NC). Obviously, the subfamilies of C, KC, and HAE with similar statistical distribution could be treated as a whole; especially as the average values of PAST_T_%, PAST_P_%, GlycoNo_T_, GlycoNo_P_, and Length_P_ were higher than any other subfamilies (Supplementary Table 5). Subfamily members of Pep were different from any other subfamily because of their short length (Supplementary Figure 1). Therefore, the threshold (55 ≤ LengthT < 90) was used to obtain Pep, and for AGPs longer than 90 amino acids, the threshold (PAST_T_% ≥ 42%) was used to discriminate C, KC, HAE, and NC with high PAST_T_% (Supplementary Figure 1). As a result, the remaining AGPs that were <42% PAST_T_ in Strategy 1 all belonged to the NC subfamily. To sum up, the results of signal peptide filtration were divided into three groups, namely the Pep subfamily was Group 1, the subfamilies of C, KC, HAE, and NC with high PAST_T_% were Group 2, and the NC with low PAST_T_% was Group 3. Moreover, the thresholds of the two variables including GlycoIndex and PAST_P_% were determined by comparing the efficiencies of these variables in identifying known AGPs (Table 5). The thresholds of GlycoIndex and PAST_P_% in identifying Pep were 0.15 and 60%, respectively. In order to obtain a strict screening threshold that could filter negative results and retain positive results at the same time, the thresholds of GlycoIndex and PAST_P_% in identifying C, KC, HAE, and NC (PAST_T_% ≥ 42) were set as 0.15 and 55%, respectively. Under these thresholds, AtAGP52 and ZmHRA1 were not detected. For the rest of the NC (PAST_T_% < 42%), the thresholds of GlycoIndex and PAST_P_% were 0.20 and 60%, respectively. Generally speaking, the glycomodules distributed throughout the AGP-like sequences of C, KC, HAE, and NC, thus the variable GlycoNo_P_ was determined to be greater than or equal to five (all AGPs in Groups 2 and 3 were greater than or equal to five glycomodules except AtAGP28). The minimums of GlycoIndex (0.13) and PAST_P_% (45%) were then used to identify subfamily members of FLA, PAG, XYLP, and homologs of known AGPs. Finally, the resultant AGP candidates were uploaded to the NCBI Batch CD-Search Tool for annotating conserved domains. EXTs and PRPs were removed according to the descriptions in Section in Materials and Methods. Subfamily classifications were performed according to the protein length, distribution patterns of AG glycomodules, and annotations of conserved domains.

**Table 5:** **The efficiencies of glycomodule index and partial PAST percentage in identifying known AGPs**.

**Group**	**Subfamily**	**GlycoIndex**				**PAST**_**P**_**%**			
		**0.10**	**0.15**	**0.20**	**0.25**	**45%**	**50%**	**55%**	**60%**
1	Pep	38	**38**^a^	37	29	38	38	38	**38**
2	C, KC, HAE, and NC^b^	67	**66**	53	23	67	66	**66**	63
3	NC^c^	10	10	**10**	7	10	10	10	**10**

a *Boldface indicates that the corresponding threshold is used in data screening of given group*.

b *Non-classical AGPs (NC) with ≥ 42% total PAST percentage*.

c *NC with < 42% total PAST percentage*.

### Identification of AGPs across plant genomes

To date, the whole genome sequences of many plant species have been released and annotated, which has enabled us to conduct bioinformatics identification of AGPs across plant genomes in one effort. After performing the consecutive operations mentioned above (Figure 2), AGP candidates were set apart from other proteins that did not meet the screening criteria (Supplementary Table 6). In tomato (*Solanum lycopersicum*), for example, signal peptide filtration and BLAST search resulted in 4317 and 258 sequences, respectively. Then the redundant sequences of the previous two steps (216) were removed and the remaining 4101 sequences of signal peptide filtration were retained for further use. Moreover, these sequences were divided into three groups based on their Length_T_ and PAST_T_%, and the parameters of the GlycoIndex and PAST_P_% were used to obtain AGP candidates. Specifically, the GlycoNo_P_ was set as five for identifying C, KC, HAE, and NC. Consequently, there were 12, 45, and 62 AGP candidates in Group 1, 2, and 3, respectively, and 130 AGP candidates in the BLAST search. Finally, a total of 249 AGP candidates were submitted to NCBI Batch CD-search for identifying annotated conserved domains. AGP candidates belonging to chimeric AGPs without fasciclin-like, plastocyanin-like, and xylogen-like domains were termed as other types of chimeric AGPs (including NC). After removing proteins belonging to EXTs and PRPs, the remaining sequences were termed as putative AGPs and mainly classified into five subfamilies, including Pep, C, KC, HAE, and chimeric AGPs.

**Figure 3:**
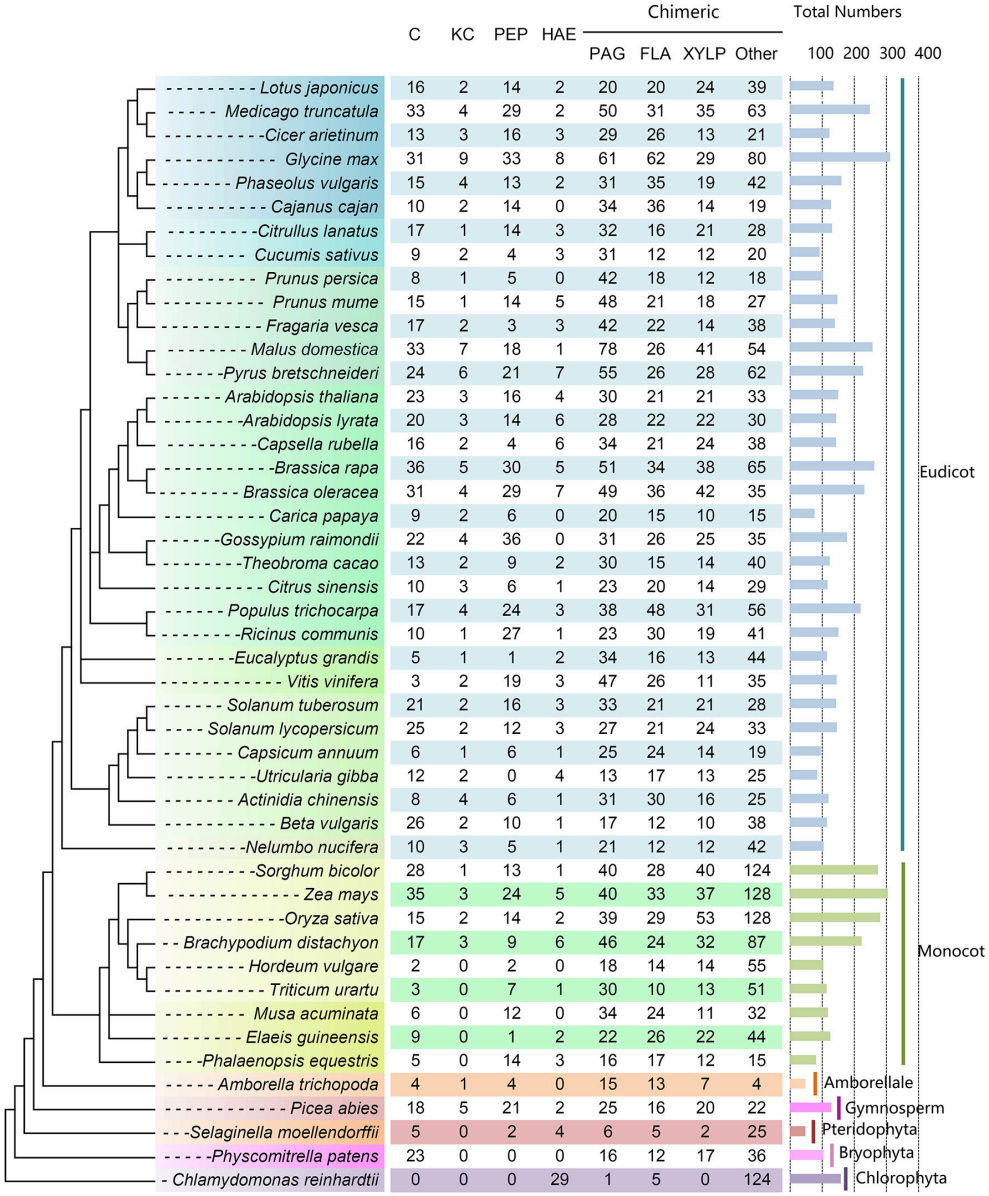
**Distribution of AGP genes across different plant lineages**. The copy number of each AGP subfamily is indicated at the top. The species tree was constructed based on the three representations of the species in Phytozome (https://phytozome.jgi.doe.gov/pz/portal.html) and then referred to the taxonomy tree in NCBI (http://www.ncbi.nlm.nih.gov/guide/taxonomy/). The horizontal bar indicates the total number of AGP genes in each plant species.

As a result, a total of 7216 putative AGPs were identified from 47 selected plant species, including 734 C, 111 KC, 597 HAE, 148 Pep, 1506 PAG, 1047 FLA, 954 XYLP, and 2092 other types of chimeric AGPs (Figure 3 and Supplementary Table 7). The number of AGPs in most plant species ranged from 100 to 200 and the number of AGPs in nine species was between 200 and 300. There were only two species that contained more than 300 AGPs (313 in *Glycine max* and 306 in *Zea mays*). The number of AGPs in seven species was <100. It was noteworthy that the AGP family members of monocots varied largely (e.g., the number of AGPs in *Sorghum bicolor*, *Z. mays*, and *O. sativa* was about three times more than in *Hordeum vulgare*). Moreover, we also found that classical AGPs, FLA, PAG, and XYLP could be found in all selected species except that classical AGPs and XYLP were absent in *Chlamydomonas reinhardtii*. Additionally, Lys-rich classical AGPs were found in all angiosperms except for several monocots (Figure 3). In particular, most AGPs (153 of 159) identified in *C. reinhardtii* were HAE and other types of chimeric AGPs. The subfamily of classical AGPs, AG-peptides, and Lys-rich classical AGPs first emerged in *Physcomitrella patens, S. moellendorffii*, and *Picea abies*, respectively.

### Exploration of new subfamilies belong to chimeric AGPs

A large number of chimeric AGPs classified as other types were obtained by the integrated screening method of AGP prediction (Supplementary Tables 7, 8). Statistical analysis of conserved domains indicated that several new subfamilies could be analogous to already classified chimeric subfamilies: FLA, PAG, and XYLP (Table 6). Typically, there were 454 chimeric AGPs with protein kinase (PK) domains in 46 species and 198 chimeric AGPs with formin homology 2 (FH2) domains in 43 species, respectively. Though the number of proteins in the other seven subfamilies was less than FLA, PAG, XYLP, and chimeric AGPs with FH2-like and PK-like domains, they exhibited a relatively higher occurrence rate than others. In general, it was found that nine kinds of chimeric AGPs existed in more than half of the 47 selected species (Table 6). Therefore, these nine kinds of chimeric AGPs were regarded as new subfamilies of chimeric AGPs in this study, including (i) chimeric AGPs with protein kinase-like domains (PK-like), (ii) chimeric AGPs with formin homology 2-like domains (FH2-like), (iii) chimeric AGPs with glycosyl hydrolase-like domains (GH-like), (iv) chimeric AGPs with pollen Ole e I-like domains (POeI-like), (v) chimeric AGPs with leucine-rich repeats-like domains (LRR-like), (vi) chimeric AGPs with X8-like domains (X8-like), (vii) chimeric AGPs with pectin methylesterase inhibitor-like domains (PMEI-like), (viii) chimeric AGPs with pectate lyase-like domains (PCL-like), and (ix) chimeric AGPs with SGNH hydrolase-like domains (SGNH-like). To further investigate the possible roles of chimeric AGPs with PK-like and FH2-like domains, the transmembrane domains of them were predicted on TMHMM Server. It was found that 93.99% (422 of 449) PK-like and 86.36% (171 of 198) FH2-like were predicted to be having at least one transmembrane motif in the middle of given sequence (Supplementary Table 7), which were compatible with their main functional aspects, such as sensing extracellular signals and transducing them into the cytoplasm.

**Table 6:** **Summary of chimeric AGPs covering more than half species**.

**Chimeric AGPs^a^**	**Abbreviations**	**Total^b^**	**Frequency^c^**
Fasciclin-like	FLA	1076	47/47
Phytocyanin-like	PAG	1506	47/47
Xylogen-like	XYLP	957	46/47
Protein kinase-like^d^	PK	449	46/47
Formin homology 2-like	FH2	198	43/47
Glycosyl hydrolase-like^e^	GH	83	41/47
Pollen Ole e I-like^f^	POeI	98	40/47
Leucine-rich repeats-like^g^	LRR	62	35/47
X8-like	X8	75	32/47
Pectin methylesterase inhibitor-like	PMEI	51	30/47
Pectate lyase-like	PCL	61	28/47
SGNH hydrolase-like	SGNH	49	26/47

a *Boldface indicates chimeric subfamilies that were previously identified in Arabidopsis and rice*.

b *Total number of each subfamily from 47 plant species*.

c *The frequency represents species number with more than one subfamily member*.

d *Chimeric AGPs with protein kinase-like domains include three types of protein kinases with conserved domains as STK_BAK1_like, STKc_IRAK, and PKc_like*.

e *Chimeric AGPs with glycosyl hydrolase-like domains include eight types of glycosyl hydrolases*.

f *Several members of chimeric AGPs with Pollen Ole e I-like domains were previously identified (AtAGP31, TTS1, and etc.)*.

g *Chimeric AGPs with leucine-rich repeats-like domains are proteins only possess LRR which are different from leucine-rich repeats receptor-like kinase (e.g., several members belong to chimeric AGPs with protein kinase-like domains)*.

To confirm that our definitions of these new subfamilies were reliable, representative protein sequences of four chimeric subfamilies in rice (*O. sativa*) were selected to show the sequence characteristics (Supplementary Figure 2), including PK-like (LOC_Os07g49240.1), FH2-like (LOC_Os02g50570.1), PCL-like (LOC_Os01g44970.1), and PMEI-like (LOC_Os07g14340.1). For chimeric subfamilies in *O. sativa*, amino acid length of members in FH2-like and PK-like subfamilies were longer than those of FLA, PAG, and XYLP. Most of the relatively longer conserved domains had a low PAST_T_% percentage (<30%) but a high PAST_P_% in their AGP-like sequences (>60%). The arrangement patterns of AG glycomodules in these AGPs were similar to known classical AGPs and chimeric AGPs (i.e., FLA, PAG, and XYLP). Typically, consecutive X-Pro repeats like “TPAPSPAPSPSP” was found in a chimeric with PK-like domain (LOC_Os07g49240.1) and there were also other discontinuous glycomodules interspaced by <10 amino acids. It was noteworthy that the GlycoIndex of these four AGPs reached universally high levels from 0.22 to 0.25. Similar glycomodule distribution patterns were also found in other chimeric AGPs not listed. All these sequences had signal peptides which indicated that they could be efficiently AG glycosylated like known AGP family members.

### Amino acid composition and phylogeny of lys-rich classical AGPs

In some classical AGPs, short Lys-rich regions (approximately 10~13 amino acids) interspaced their PAST-rich regions, which were termed as Lys-rich classical AGPs (Sun et al., 2005). For example, LeAGP1, the first Lys-rich classical AGP identified from tomato (*S. lycopersicum* formerly *Lycopersicon esculentum*), contained a short Lys-rich sequence “KGKVKGKKGKKHN” (Li and Showalter, 1996). However, the Lys-rich region of AtAGP19 “KHKRKHKHKRHHH” was not only rich in Lys but also with a high percentage of His (i.e., five Lys, six His, and two Arg; Supplementary Table 9). It was noteworthy that the other eight residues were basic amino acids which are polar and positively charged. Identification of Lys-rich AGPs in 47 plants made it possible to confirm whether the occurrence of His and Arg residues was a special case only found in AtAGP19. Therefore, we statistically analyzed the length of Lys-rich regions and the number of Lys, His, Arg, and other amino acid residues. As shown in Figure 3 and Supplementary Table 9, Lys-rich AGPs were present in 39 species but absent in eight species. For the short Lys-rich regions, lengths were largely variable from 5 to 22 amino acids. Moreover, the amino acid composition of the short Lys-rich regions was considerably different. The Lys-rich, His-rich, or Arg-rich regions were characterized by three kinds of basic amino acids which contributed the most to the short regions. Typically, 72 Lys-rich regions were found in 111 proteins, followed by 24 His-rich regions and four Arg-rich regions. Also, we found eight Lys/His-rich regions (i.e., Lys and His were equal in number) and three His/Arg-rich regions (i.e., His and Arg were equal in number), respectively, but did not find any Lys/Arg-rich region in this study. Lys residues were absent in the basic amino acid-rich region of Glyma.02G130000.1.p and only one Lys residue was found in the short region (14 amino acids) of Glyma.01G092300.1.p. More specifically, it would be inappropriate if calling AGPs with few and even without Lys residues as “Lys-rich AGPs.”

**Figure 4:**
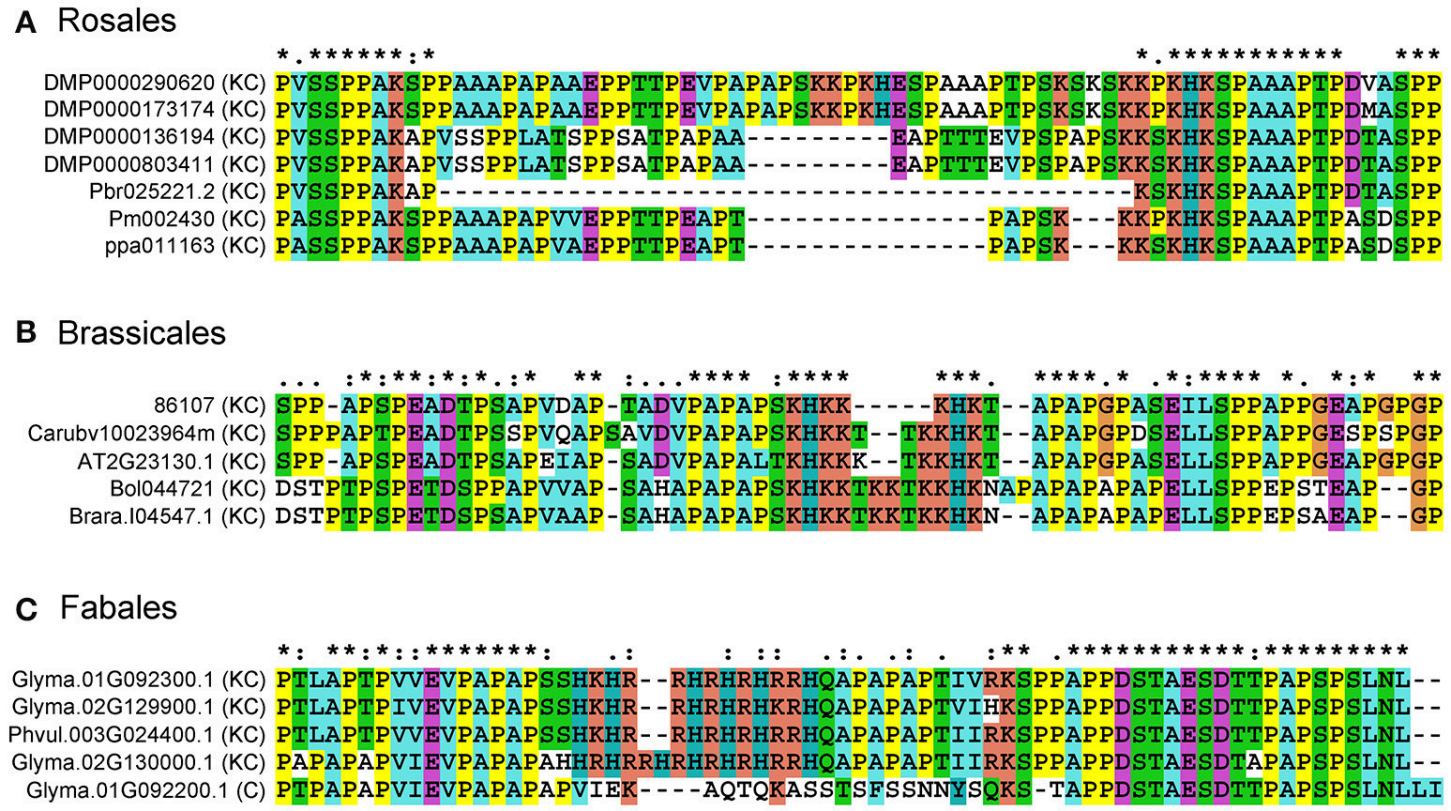
**Multiple sequence alignments of representatives from classical and Lys-rich classical AGPs**. Amino acid sequences were aligned using the Clustal X 1.83 program with default parameters to show the deletion/insertion model amongst classical and Lys-rich classical AGPs. **(A)** Lys-rich classical AGPs from Rosales; **(B)** Lys-rich classical AGPs from Brassicales; **(C)** classical and Lys-rich classical AGPs from Fabales. The asterisk (^*^), colon (:), and dot (.) represent different conservative level from high to low.

To understand the sequence variation of Lys-rich AGPs, multiple sequence alignments were performed using representative Lys-rich AGPs from Rosales, Brassicales, and Fabales species. The length of Lys-rich regions was varied in closely related Rosales Lys-rich AGPs. For instance, two short Lys-rich regions were found in both DMP0000290620 and DMP0000173174, namely “KKPKH” and “KSKSKKPKHK” were spaced by “ESPAAAPTPS.” However, in Pm002430 and ppa011163, the “KKPKH” was missed and “KSKSKKPKHK” was shortened into “KKKPKHK” and “KKKSKHK,” respectively. Moreover, the Lys-rich region was only a five-amino acid sequence “KSKHK” in Pbr025221.2 (Figure 4). Similar patterns were found in representatives of Brassicales, there were seven amino acids “KHKKKHK,” ten amino acids “KHKKKTKKHK,” and twelve amino acids “KHKKTKKTKKHK” in the Lys-rich regions. Interestingly in Fables, we not only found the sequence length change of Lys-rich regions between homologs but also found the Lys-rich region was absent in Glyma.01G092200.1, meaning that the Lys-rich AGPs had a homologous classical AGP.

**Figure 5:**
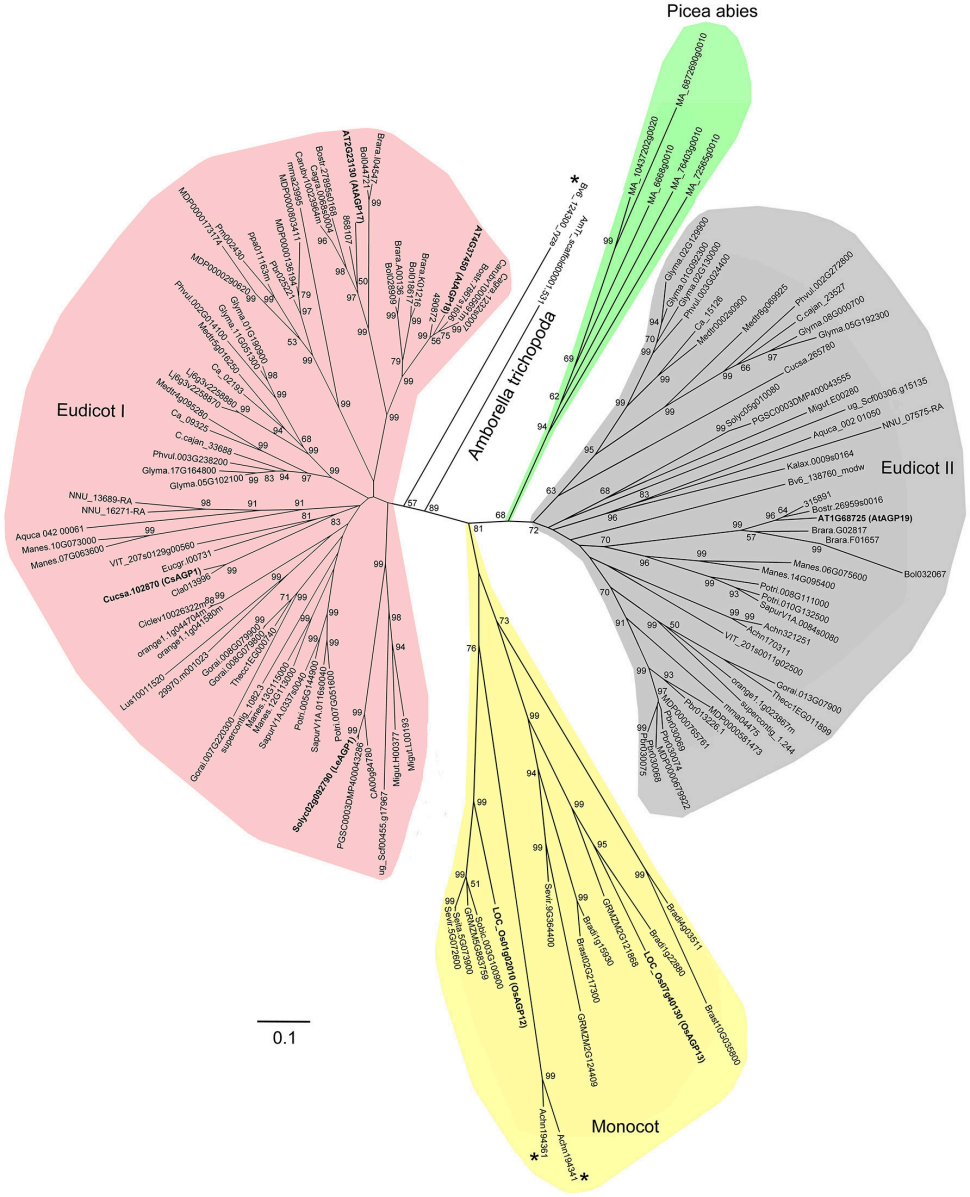
**Phylogenetic relationship of Lys-rich AGPs**. The phylogenetic tree is constructed based on the multiple sequence alignments of Lys-rich AGPs using MEGA 6.0. Bootstrapping is performed from 1000 reiterations of the neighbor-joining method. Scale bar represents 0.1 amino acid substitutions per site.

In order to investigate the evolutionary events of Lys-rich AGPs, we also identified the Lys-rich AGPs of 16 angiosperms (especially monocot species) and Chlorophyta representatives in addition to the 47 plant species. To sum up, a total of 138 Lys-rich AGPs were identified (Supplementary Table 10). It was clear that the Lys-rich AGPs were only present in seed plants (Spermatophyta) including gymnosperm and angiosperm but absent in spikemoss (*S. moellendorffii*), moss (*P. patens*), and green alga (Chlamydomonadales). Besides, several monocot species were also lacking Lys-rich AGPs. Moreover, the full-length amino acid sequences of 138 Lys-rich AGPs were used to perform multiple sequence alignment and generate phylogenetic trees. The phylogeny analysis suggested that the Lys-rich AGPs experienced at least two ancient duplications which gave rise to the three subgroups: monocot group, eudicot group I, and eudicot group II (Figure 5). The Lys-rich AGPs from the distinctive angiosperm plant−*Amborella trichopoda* and the gymnosperm plant *P. abies* were closer to eudicot group I and II, respectively. There were three exceptions, *Beta vulgaris* (Bv6_124300_ryze.t1) was independent of the eudicot group I and another two from *Actinidia chinensis* (Achn194341 and Achn194361) were in the monocot group (Figure 5).

## Discussion

### The development of AGP prediction: from amino acid bias and BIO OHIO to finding-AGP

Following the complete genome sequencing of many plant species, protein sequences were annotated according to their sequence homology with other proteins. However, for AGP family members, most were incorrectly annotated due to their relatively low homology. Therefore, in order to annotate and identify the entire AGP family, we must find an accurate and efficient method to separate them from other proteins. The amino acid bias method (i.e., sequences are biased for Pro, Ala, Ser, and Thr) was firstly proposed to identify AGPs (Schultz et al., 2002), which efficiently identified 62 candidate genes from 25,617 protein sequences with a high PAST_T_% in *Arabidopsis* (i.e., >50%). However, most members of several subfamilies were absent, including Pep, chimeric AGPs (FLA, PAG, and XYLP), and NC. If the threshold of PAST_T_% was reduced to 30% to cover most of the missed AGPs mentioned above, many false positive results were found. Finally, Schultz et al., 2002 discussed the “windows” approach to identify AGP-like sequences by calculating the PAST percentage in “windows” of 15–25 amino acid residues, which could reduce false positives when the PAST_T_% was relatively low. Recently, the sliding window feature was developed in the BIO OHIO program (Showalter et al., 2010). When the PAST percentage was >60% in window size of 10 amino acids, a large number of false positives were generated and limited the use of the sliding windows feature.

In this study, we compiled a Python script called Finding-AGP to find and analyze AGP-like sequences. Compared to the sliding window feature of BIO OHIO, the most distinctive difference in Finding-AGP was to extract AGP-like sequences based on the definition of effective AG glycosylation instead of specifying the length of a user-defined window. Based on the statistical analysis of known AGPs, the glycomodule patterns were designated as Ala-Pro, Pro-Ala, Ser-Pro, Pro-Ser, Thr-Pro, and Pro-Thr. Seven continuous variables (Length_T_, PAST_T_%, GlycoNo_T_, Length_P_, PAST_P_%, GlycoNo_P_, and GlycoIndex) and an dichotomic variable (whether there was a predicted signal peptide) were proposed and incorporated into the Finding-AGP program (Table 3). An integrated strategy proposed in this study could separately identify different subfamilies with reduced false positives. The Finding-AGP program had obvious advantages in speed and accuracy, especially in identification of chimeric AGPs with low PAST_T_% (<42%), the number of sequences from most species was <200 (44 of 47 species, Supplementary Table 6). Though it was believed that we identified the absolute majority of all AGPs, some “fishes may have escaped from the net.” Specifically, because of our searching criteria, proteins without a signal peptide and at the same time with low homology (*e*-value > *e*^−3^) could not be obtained. Also, the relatively high threshold settings in screening NC with more than 42% PAST_T_ may have led to missing some putative AGPs.

### Evolutionary history of the AGP gene family

Many previous studies of AGPs start with phrases like “AGPs were widely distributed in higher plants [plants, plant kingdom, etc.]” (van Hengel and Roberts, 2003; Gaspar et al., 2004; Qin and Zhao, 2006). In fact, the distribution patterns of AGPs were mostly based on 22 species with known AGP genes and other species by the detection of AGP carbohydrate epitopes. In this study, the identification of AGP genes in 47 plant species enabled us to investigate the evolutionary history of this gene family. It is noteworthy that five FLA, one PAG and several proteins with AGP-like and EXT-like glycomodules (e.g., continuous SP or SP_2−4_ repeats) were found in green alga, indicated that other subfamilies (i.e., C, KC, Pep, and XYLP) might have emerged after the divergence of the green alga and land plants. Previously, it was reported that Xylogen (a member of XYLP) mediated the transformation of undifferentiated suspension cells in liquid culture medium into tracheary elements, which are a basal component in the xylem of vascular tissue (Motose et al., 2004). We speculated that the occurrence of XYLP might be a key incident in the evolution of vascular plants from lower plants, namely the formation of main components of vascular bundles (tracheary elements) could take place in unicellular or multicellular green alga. Corresponding to this point of view, the XYLP members first emerged in moss and existed in all vascular plants (Figure 3). Moreover, regarding the close-related three subfamilies (C, KC, and Pep), C was first appeared in *P. patens* and then Pep in *S. moellendorffii*, and at last KC in *P. abies*.

The origin of KC emergence needs to be further elucidated, but the most likely scenario is that it took place after the divergence of *S. moellendorffii* and the common ancestor of seed plants. Moreover, based on the occurrence order of C and KC, the KC was most likely produced by insertions of Lys codons in the coding sequences of C. There were two phylogenetic branches in eudicots but only one for monocot, and KC was absent in several monocots, implying a substantially different evolutionary fate between monocots and eudicots. The difference was probably happened in the ancient whole genome duplication (WGD) event that occurred when the ancestors of angiosperms generated duplicates of each gene (Jiao et al., 2011, 2014). However, these speculations need to be testified by adding more species of Gymnosperm, Lycopodiophyta, and Bryophyta to improve the resolution of the phylogenetic trees.

### The diversity of AGP gene family

In this study, a large number of other types of chimeric AGPs were identified along with known subfamilies, which contributed to almost half of the total number. However, whether the presence of glycomodules in newly identified chimeric AGPs led to successful AG glycosylation should be verified in the future. Up to now, the experimental evidences of AG glycosylation were only proved in several members of FLA, PAG, XYLP, and chimeric AGPs with POeI-like domains (Johnson et al., 2003; Mashiguchi et al., 2004; Hijazi et al., 2012). The major challenge will be to discover a high throughput method to verify the post-translational modifications of putative chimeric AGPs obtained by bioinformatics predictions.

The existence of many HAE indicated that there was not an insurmountable gap between AGPs and EXTs in the course of plant evolution. The pollen extensin-like 1 (PEX1) and LRX members related to the pollen tube and root growth had already been reported in several studies (Rubinstein et al., 1995; Baumberger et al., 2001; Draeger et al., 2015) and the LRX members in rice and *Arabidopsis* were also identified (Baumberger et al., 2003). Previous studies about EXTs summarized the existence of formin-like EXTs, proline-rich extensin-like receptor kinases (PERKs), and leucine-rich-repetitive EXTs (LRXs; Borassi et al., 2016). AG glycomodules were also found in many formins, receptor-like kinases, and proteins with leucine-rich-repetitive (LRR) motifs. These evidences led us to believe that three subfamilies of chimeric AGPs (PK-like, LRR-like, and FH2-like) were most likely existed. Moreover, at least eight chimeric AGPs with Pollen_Ole_e_I domains were previously identified, four of which were proved to be AG glycosylated, including TTS1, TTS2, DcAGP1, and AtAGP31 (Cheung et al., 1995; Wu et al., 1995; Baldwin et al., 2001; Liu and Mehdy, 2007; Hijazi et al., 2014b). We found a total of 98 chimeric AGPs with POeI-like domains across 40 plant species (Table 6). In addition, the PMEI-like (HAE1), LAM (AtAGP31) alpha_CA_prokaryotic_like (AtAGP33) were also identified (Liu and Mehdy, 2007; Showalter et al., 2010). According to an uncompleted statistic, more than 100 conserved domains from the NCBI CDD website were found in putative AGPs identified in this study (Supplementary Table 7), only a dozen of them were introduced and the rest of them needed to be further investigated.

The diversity of the AGP gene family not only resulted from the various kinds of subfamilies but also relied on the variable gene numbers in each subfamily. In green algae, although 159 total AGP-like sequences were found, the subfamilies of C, KC, and Pep were absent, simple repeats were typical characteristics of these sequences (e.g., [SP]n and [SP_2−4_]n). C and Pep appeared in Bryophyta (*P. patens*) and Pteridophyta (*S. moellendorffii*), respectively (Figure 3). The AGPs flourished in both subfamilies and numbers in seed plants (gymnosperm and angiosperm) except for the decreased numbers in several species. The incidents of AGP molecular evolution needs to be elucidated in the future.

Thirdly, the sequences of AGPs were diverse even in the same subfamily. Building phylogenetic trees failed in classical AGPs because of the low sequence homology, but we could generate a phylogenetic tree of Lys-rich AGPs even if it was largely divergent (Figure 5). We only observed high sequence homology in closely related species (e.g., Brassicales). Moreover, the Lys-rich domains were also different in length and amino acid constitution (Supplementary Table 9). More specifically, Lys was actually abundant in most Lys-rich AGPs, but His-rich and Arg-rich AGPs were also found. The short regions were usually rich in the three basic amino acids; thus it would be more appropriate if this subfamily was named basic amino acid-rich AGPs.

### Roles of chimeric AGPs in modulating cell wall mechanics and mediating signaling between the cell wall and cytoplasm

**Figure 6:**
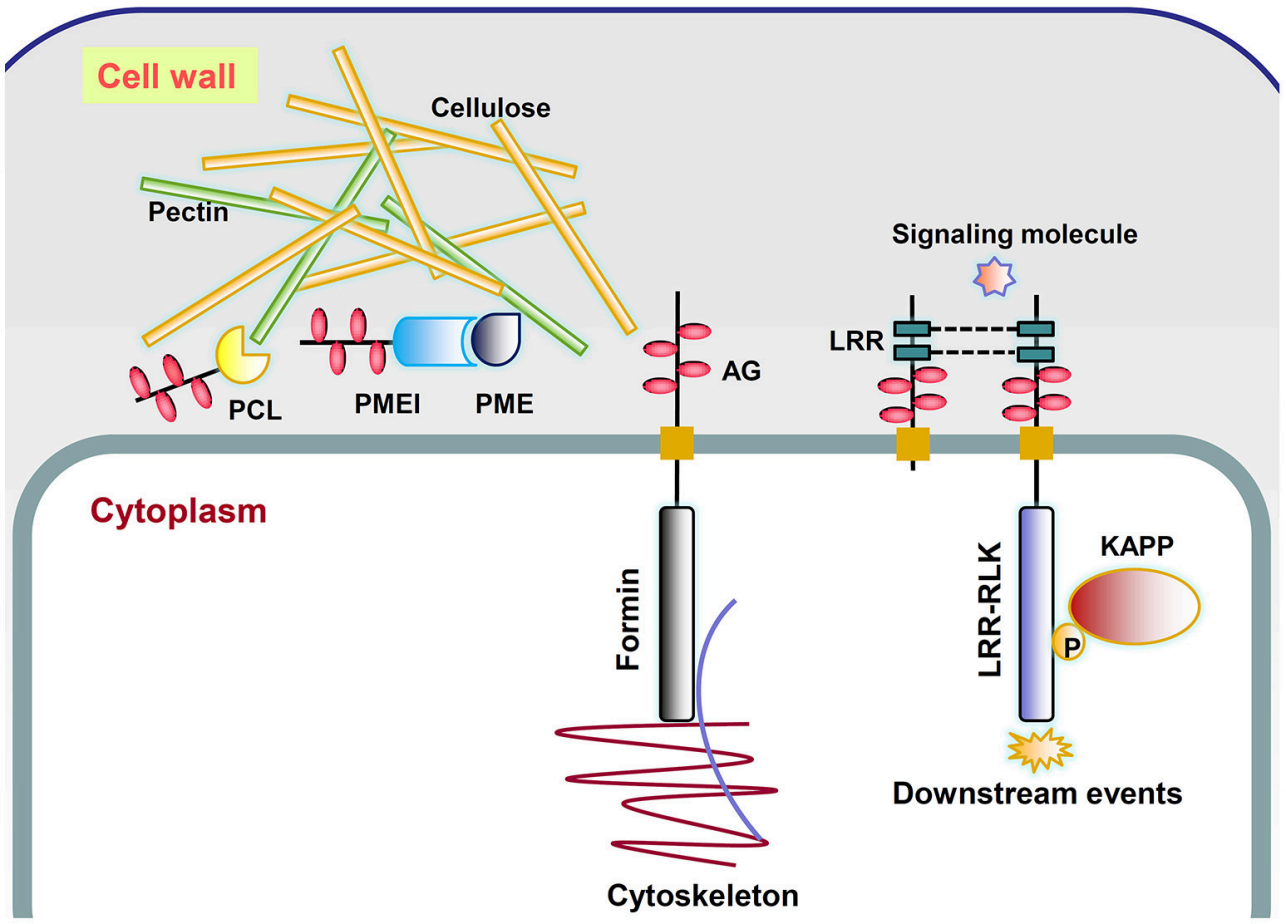
**Putative working model of several types of chimeric AGPs**. PCL, chimeric AGPs with pectin lyase-like domain; PME, pectin methylesterase; PMEI, chimeric AGPs with pectin methylesterase inhibitor-like domain; LRR, chimeric AGPs with leucine-rich repeats-like domain; Formin, chimeric AGPs with formin homology 2-like domain; LRR-RLK, chimeric AGPs with leucine-rich repeat receptor kinase-like domain; AG, arabinogalactan, which is represented by pink ovals; P, phosphate (yellow circle); KAPP, kinase-associated protein phosphatase.

The fact that AGPs could specifically bind to the β-Yariv reagent suggested that they might interact with β-linked polysaccharides in the cell wall matrix (Kitazawa et al., 2013; Hijazi et al., 2014b). The co-purification of Yariv reactive glycoproteins and cellulose also indicated possible roles of AGPs in cell wall mechanics (Girault et al., 2000). A classical AGP named ARABINOXYLAN PECTIN ARABINOGALACTAN PROTEIN1 (APAP1) was proved to be covalently attached to wall matrix hemicellulosic and pectic polysaccharides through the rhamnosyl residue arabinogalactan (AG) in *Arabidopsis thaliana* (Tan et al., 2013). Besides, it was also found that AGP31 interacted with rhamnogalacturonan I (RGI) through its PRP-AGP containing Cys (PAC) domain and bound methylesterified polygalacturonic acid through its His-stretch (Hijazi et al., 2014b). In this study, a large number of genes encoding X8 proteins and glycosyl hydrolases with AGP-like glycomodules were found, indicating possible roles of AGPs in binding to carbohydrates and catalyzing cell wall polymer biosynthesis. AGPs might be a “pectin plasticizer” based on the observations of porosity when AGPs are incorporated into pectin gel (Lamport and Kieliszewski, 2005). In addition, a tight association of type II AG with pectin has been observed and reviewed by Nothnagel (1997). Our study has indicated that obtaining experimental evidences between AGPs and pectin would be a useful direction. Three kinds of chimeric AGPs related to pectin were discovered including pectate lyase, pectin methylesterase inhibitor, and pectin esterase, which might lead to explanations of AGP function in rapid tip growth and pathogen infection (Mollet et al., 2002; Vorwerk et al., 2004; Figure 6).

It was previously found that the addition of β-Yariv reagent inhibited cell division of a rose cell suspension line (Serpe and Nothnagel, 1994) and also interrupted the first zygotic asymmetric division of tobacco (Qin and Zhao, 2006). Moreover, β-Yariv treatment rapidly disrupted the normal organization of the microtubule and actin cytoskeleton in tobacco BY2 cells (Sardar et al., 2006). All these reports indicated that the irregular divisions were accompanied by cytoskeleton change. The change of cytokinesis and cell polarization is controlled by the rearrangement of the actin and microtubule cytoskeleton, which might be related to the functions of chimeric AGPs with formin homology 2-like domains (Figure 6).

The developmental roles of AGPs made us believe that they might interact with receptor-like kinases (RLKs) and act as a co-receptor (Zhang et al., 2011). This flow of ideas considered that AGPs might be involved in phytohormone and stress signaling as co-receptors because they lacked receptor-like sequences. For example, β-Yariv treatment of barley possibly acted in gibberellic acid signal transduction through suppressing the induction of α-amylase enzyme to regulate starch degradation (Mashiguchi et al., 2008). Immunofluorescence of AG epitopes in tobacco protoplasts revealed their co-localization patterns with wall associated kinase (WAK; Gens et al., 2000). Hypotheses about this phenomenon focus on the interaction between AGP and WAKs. However, Seifert and Roberts (2007) found that ~60 out of 620 RLK proteins had putative AG glycomodules. If RLKs could be part of the AGP family, finding receptors that interacted with AGP (deemed as co-receptor) was heading in the wrong direction. In this study, a total of 454 chimeric AGPs with protein kinase-like domains were identified across 46 plant species except *A. trichopoda*. The subgroups of STK_BAK1_like and STKc_IRAK were possible receptor-like candidates of AGPs. *A. thaliana* BRASSINOSTEROID (BR) INSENSITIVE 1 (BRI1), the receptor for BRs, belonged to STKc_IRAK group (Li et al., 2002). A total of 229 STK_BAK1_like and STKc_IRAK chimeric AGPs were identified across 44 plant species except *A*. *trichopoda, C. reinhardtii*, and *Elaeis guineensis*. RLK proteins consist of a Pro-rich extracellular domain, transmembrane region, and an intracellular kinase domain (Figure 6). They might be connected with the cell wall and function in sensing signals from the cell wall to cytoplasm by activating their kinase domains (Silva and Goring, 2002; Goring, 2015; Humphrey et al., 2015). The LRR motifs with the consensus sequence LxxLxLxxN/CxL were not only found in receptor-like kinases (RLKs; e.g., BRI1) but also found in many chimeric AGPs (called LRR-like; Kobe and Kajava, 2001). The roles of the LRR domain were highlighted in protein–protein interactions which might bind to extracellular ligands or form dimers. On the other hand, the molecular functions of AGPs involved in signaling were assumed to depend on the bonds of glucuronic acid residues of AG and Ca^2+^ (Lamport and Várnai, 2013). The recycling of Ca^2+^ was proposed to be conducted by an AGP-Ca^2+^ oscillator, which provide suitable explanations for the involvement of AGPs in Ca^2+^ and auxin-related plant morphogenesis (Lamport et al., 2014).

## Author contributions

HM and QC conceived and designed the research plans; YM and CY performed most of the experiments and analyzed the data; HL, WW, YL, and YW provided technical assistance to YM and CY; YM wrote the article with contributions of all the authors; HM and QC supervised and complemented the writing.

### Conflict of interest statement

The authors declare that the research was conducted in the absence of any commercial or financial relationships that could be construed as a potential conflict of interest.

## References

[B1] Baldwin T. C. Domingo C. Schindler T. Seetharaman G. Stacey N. Roberts K. (2001). DcAGP1, a secreted arabinogalactan protein, is related to a family of basic proline-rich proteins. Plant Mol. Biol. 45, 421–435. 10.1023/A:1010637426934 11352461

[B2] Baumberger N. Doesseger B. Guyot R. Diet A. Parsons R. L. Clark M. A.. (2003). Whole-genome comparison of leucine-rich repeat extensins in Arabidopsis and rice. A conserved family of cell wall proteins form a vegetative and a reproductive clade. Plant Physiol. 131, 1313–1326. 10.1104/pp.102.014928 12644681 PMC166891

[B3] Baumberger N. Ringli C. Keller B. (2001). The chimeric leucine-rich repeat/extensin cell wall protein LRX1 is required for root hair morphogenesis in *Arabidopsis thaliana*. Genes Dev. 15, 1128–1139. 10.1101/gad.200201 11331608 PMC312681

[B4] Borassi C. Sede A. R. Mecchia M. A. Salgado Salter J. D. Marzol E. Muschietti J. P.. (2016). An update on cell surface proteins containing extensin-motifs. J. Exp. Bot. 67, 477–487. 10.1093/jxb/erv455 26475923

[B5] Borner G. H. Lilley K. S. Stevens T. J. Dupree P. (2003). Identification of glycosylphosphatidylinositol-anchored proteins in arabidopsis. A proteomic and genomic analysis. Plant Physiol. 132, 568–577. 10.1104/pp.103.021170 12805588 PMC166998

[B6] Cheung A. Y. Wang H. Wu H. M. (1995). A floral transmitting tissue-specific glyxoprotein attracts pollen tubes and stimulates their growth. Cell 82, 383–393. 10.1016/0092-8674(95)90427-1 7634328

[B7] Draeger C. Ndinyanka Fabrice T. Gineau E. Mouille G. Kuhn B. M. Moller I.. (2015). Arabidopsis leucine-rich repeat extensin (LRX) proteins modify cell wall composition and influence plant growth. BMC Plant Biol. 15:155. 10.1186/s12870-015-0548-8 26099801 PMC4477543

[B8] Ellis M. Egelund J. Schultz C. J. Bacic A. (2010). Arabinogalactan-proteins: key regulators at the cell surface? Plant Physiol. 153, 403–419. 10.1104/pp.110.156000 20388666 PMC2879789

[B9] Faik A. Abouzouhair J. Sarhan F. (2006). Putative fasciclin-like arabinogalactan-proteins (FLA) in wheat (*Triticum aestivum*) and rice (*Oryza sativa*): identification and bioinformatic analysis. Mol. Genet. Genomics 276, 478–494. 10.1007/s00438-006-0159-z 16944204

[B10] Gaspar Y. Johnson K. L. McKenna J. A. Bacic A. Schultz C. J. (2001). The complex structures of arabinogalactan-proteins and the journey towards understanding function. Plant Mol. Biol. 47, 161–176. 10.1023/A:1010683432529 11554470

[B11] Gaspar Y. Nam J. Schultz C. J. Lee L. Y. Gilson P. R. (2004). Characterization of the Arabidopsis lysine-rich arabinogalactan-protein AtAGP17 mutant (rat1) that results in a decreased efficiency of agrobacterium transformation. Plant Physiol. 135, 2162–2171. 10.1104/pp.104.045542 15286287 PMC520787

[B12] Gens J. S. Fujiki M. Pickard B. G. (2000). Arabinogalactan protein and wall-associated kinase in a plasmalemmal reticulum with specialized vertices. Protoplasma 212, 115–134. 10.1007/BF01279353 11543565

[B13] Gilson P. Gaspar Y. M. Oxley D. Youl J. J. Bacic A. (2001). NaAGP4 is an arabinogalactan-protein whose expression is suppressed by wounding and fungal infection in *Nicotiana alata*. Protoplasma 215, 128–139. 10.1007/BF01280309 11732052

[B14] Girault R. His I. Andeme-Onzighi C. Driouich A. Morvan C. (2000). Identification and partial characterization of proteins and proteoglycans encrusting the secondary cell walls of flax fibres. Planta 211, 256–264. 10.1007/s004250000281 10945220

[B15] Goring D. R. (2015). PERK-KIPK-KCBP signalling negatively regulates root growth in *Arabidopsis thaliana*. J. Exp. Bot. 66, 71–83. 10.1093/jxb/eru390 25262228 PMC4265151

[B16] Griffiths J. S. Crepeau M. J. Ralet M. C. Seifert G. J. North H. M. (2016). Dissecting seed mucilage adherence mediated by FEI2 and SOS5. Front. Plant Sci. 7:1073. 10.3389/fpls.2016.01073 27524986 PMC4965450

[B17] Hijazi M. Durand J. Pichereaux C. Pont F. Jamet E. Albenne C. (2012). Characterization of the arabinogalactan protein 31 (AGP31) of *Arabidopsis thaliana*: new advances on the Hyp-O-glycosylation of the Pro-rich domain. J. Biol. Chem. 287, 9623–9632. 10.1074/jbc.M111.247874 22270363 PMC3308734

[B18] Hijazi M. Roujol D. Nguyen-Kim H. Del Rocio Cisneros Castillo L. Saland E. Jamet E.. (2014b). Arabinogalactan protein 31 (AGP31), a putative network-forming protein in *Arabidopsis thaliana* cell walls? Ann. Bot. 114, 1087–1097. 10.1093/aob/mcu038 24685714 PMC4195544

[B19] Hijazi M. Velasquez S. M. Jamet E. Estevez J. M. Albenne C. (2014a). An update on post-translational modifications of hydroxyproline-rich glycoproteins: toward a model highlighting their contribution to plant cell wall architecture. Front. Plant Sci. 5:395. 10.3389/fpls.2014.00395 25177325 PMC4132260

[B20] Humphrey T. V. Haasen K. E. Aldea-Brydges M. G. Sun H. Zayed Y. Indriolo E.. (2015). PERK-KIPK-KCBP signalling negatively regulates root growth in *Arabidopsis thaliana*. J. Exp. Bot. 66, 71–83. 10.1093/jxb/eru390 25262228 PMC4265151

[B21] Jiao Y. Li J. Tang H. Paterson A. H. (2014). Integrated syntenic and phylogenomic analyses reveal an ancient genome duplication in monocots. Plant Cell 26, 2792–2802. 10.1105/tpc.114.127597 25082857 PMC4145114

[B22] Jiao Y. Wickett N. J. Ayyampalayam S. Chanderbali A. S. Landherr L. Ralph P. E.. (2011). Ancestral polyploidy in seed plants and angiosperms. Nature 473, 97–100. 10.1038/nature09916 21478875

[B23] Johnson K. L. Jones B. J. Bacic A. Schultz C. J. (2003). The fasciclin-like arabinogalactan proteins of Arabidopsis. A multigene family of putative cell adhesion molecules. Plant Physiol. 133, 1911–1925. 10.1104/pp.103.031237 14645732 PMC300743

[B24] Kavi Kishor P. B. Hima Kumari P. Sunita M. S. Sreenivasulu N. (2015). Role of proline in cell wall synthesis and plant development and its implications in plant ontogeny. Front. Plant Sci. 6:544. 10.3389/fpls.2015.00544 26257754 PMC4507145

[B25] Kitazawa K. Tryfona T. Yoshimi Y. Hayashi Y. Kawauchi S. Antonov L.. (2013). β-galactosyl Yariv reagent binds to the β-1,3-galactan of arabinogalactan proteins. Plant Physiol. 161, 1117–1126. 10.1104/pp.112.211722 23296690 PMC3585584

[B26] Kobayashi Y. Motose H. Iwamoto K. Fukuda H. (2011). Expression and genome-wide analysis of the xylogen-type gene family. Plant Cell Physiol. 52, 1095–1106. 10.1093/pcp/pcr060 21558309

[B27] Kobe B. Kajava A. V. (2001). The leucine-rich repeat as a protein recognition motif. Curr. Opin. Struct. Biol. 11, 725–732. 10.1016/S0959-440X(01)00266-4 11751054

[B28] Lamport D. T. Kieliszewski M. J. (2005). Stress upregulates periplasmic arabinogalactan proteins. Plant Biosyst. 139, 60–64. 10.1080/11263500500055106 16411951

[B29] Lamport D. T. A. Várnai P. (2013). Periplasmic arabinogalactan glycoproteins act as a calcium capacitor that regulates plant growth and development. New Phytol. 197, 58–64. 10.1111/nph.12005 23106282

[B30] Lamport D. T. Varnai P. Seal C. E. (2014). Back to the future with the AGP-Ca2+ flux capacitor. Ann. Bot. 114, 1069–1085. 10.1093/aob/mcu161 25139429 PMC4195563

[B31] Li J. Gao G. Zhang T. Wu X. (2013). The putative phytocyanin genes in Chinese cabbage (*Brassica rapa* L.): genome-wide identification, classification and expression analysis. Mol. Genet. Genomics 288, 1–20. 10.1007/s00438-012-0726-4 23212439

[B32] Li J. Wen J. Lease K. A. Doke J. T. Tax F. E. Walker J. C. (2002). BAK1, an Arabidopsis LRR receptor-like protein kinase, interacts with BRI1 and modulates brassinosteroid signaling. Cell 110, 213–222. 10.1016/S0092-8674(02)00812-7 12150929

[B33] Li S. X. Showalter A. M. (1996). Cloning and developmental/stress-regulated expression of a gene encoding a tomato arabinogalactan protein. Plant Mol. Biol. 32, 641–652. 10.1007/BF00020205 8980516

[B34] Liu C. Mehdy M. C. (2007). A nonclassical arabinogalactan protein gene highly expressed in vascular tissues, AGP31, is transcriptionally repressed by methyl jasmonic acid in Arabidopsis. Plant Physiol. 145, 863–874. 10.1104/pp.107.102657 17885091 PMC2048811

[B35] Ma H. Zhao J. (2010). Genome-wide identification, classification, and expression analysis of the arabinogalactan protein gene family in rice (*Oryza sativa* L.). J. Exp. Bot. 61, 2647–2668. 10.1093/jxb/erq104 20423940 PMC2882264

[B36] Ma H. Zhao H. Liu Z. Zhao J. (2011). The phytocyanin gene family in rice (*Oryza sativa* L.): genome-wide identification, classification and transcriptional analysis. PLoS ONE 6:e25184. 10.1371/journal.pone.0025184 21984902 PMC3184959

[B37] Ma T. Ma H. Zhao H. Qi H. Zhao J. (2014). Identification, characterization, and transcription analysis of xylogen-like arabinogalactan proteins in rice (*Oryza sativa* L.). BMC Plant Biol. 14:299. 10.1186/s12870-014-0299-y 25407280 PMC4239379

[B38] MacMillan C. P. Taylor L. Bi Y. Southerton S. G. Evans R. Spokevicius A. (2015). The fasciclin-like arabinogalactan protein family of *Eucalyptus grandis* contains members that impact wood biology and biomechanics. New Phytol. 206, 1314–1327. 10.1111/nph.13320 25676073

[B39] Mashiguchi K. Asami T. Suzuki Y. (2009). Genome-wide identification, structure and expression studies, and mutant collection of 22 early nodulin-like protein genes in Arabidopsis. Biosci. Biotechnol. Biochem. 73, 2452–2459. 10.1271/bbb.90407 19897921

[B40] Mashiguchi K. Urakami E. Hasegawa M. Sanmiya K. Matsumoto I. Yamaguchi I.. (2008). Defense-related signaling by interaction of arabinogalactan proteins and beta-glucosyl Yariv reagent inhibits gibberellin signaling in barley aleurone cells. Plant Cell Physiol. 49, 178–190. 10.1093/pcp/pcm175 18156132

[B41] Mashiguchi K. Yamaguchi I. Suzuki Y. (2004). Isolation and identification of glycosylphosphatidylinositol-anchored arabinogalactan proteins and novel β-glucosyl Yariv-reactive proteins from seeds of rice (*Oryza sativa*). Plant Cell Physiol. 45, 1817–1829. 10.1093/pcp/pch208 15653800

[B42] Mollet J. C. Kim S. Jauh G. Y. Lord E. M. (2002). Arabinogalactan proteins, pollen tube growth, and the reversible effects of Yariv phenylglycoside. Protoplasma 219, 89–98. 10.1007/s007090200009 11926071

[B43] Motose H. Sugiyama M. Fukuda H. (2004). A proteoglycan mediates inductive interaction during plant vascular development. Nature 429, 873–878. 10.1038/nature02613 15215864

[B44] Nguema-Ona E. Vicré-Gibouin M. Gotté M. Plancot B. Lerouge P. Bardor M.. (2014). Cell wall O-glycoproteins and N-glycoproteins: aspects of biosynthesis and function. Front. Plant Sci. 5:499. 10.3389/fpls.2014.00499 25324850 PMC4183102

[B45] Nothnagel E. A. (1997). Proteoglycans and related components in plant cells. Int. Rev. Cytol. 174, 195–291. 10.1016/S0074-7696(08)62118-X 9161008

[B46] Petersen T. N. Brunak S. von Heijne G. Nielsen H. (2011). SignalP 4.0: discriminating signal peptides from transmembrane regions. Nat. Methods 8, 785–786. 10.1038/nmeth.1701 21959131

[B47] Qin Y. Zhao J. (2006). Localization of arabinogalactan proteins in egg cells, zygotes, and two-celled proembryos and effects of beta-D-glucosyl Yariv reagent on egg cell fertilization and zygote division in *Nicotiana tabacum* L. J Exp. Bot. 57, 2061–2074. 10.1093/jxb/erj159 16720612

[B48] Rubinstein A. L. Marquez J. Suarez-Cervera M. Bedinger P. A. (1995). Extensin-like glycoproteins in the maize pollen tube wall. Plant Cell 7, 2211–2225. 10.1105/tpc.7.12.2211 12242372 PMC161074

[B49] Sardar H. S. Yang J. Showalter A. (2006). Molecular interactions of arabinogalactan-proteins (AGPs) with cortical microtubules and F-actin in bright yellow-2 (BY-2) tobacco cultured cells. Plant Physiol. 142, 1469–1479. 10.1104/pp.106.088716 17056757 PMC1676048

[B50] Schultz C. J. Johnson K. L. Currie G. Bacic A. (2000). The classical arabinogalactan protein gene family of Arabidopsis. Plant Cell 12, 1751–1768. 10.1105/tpc.12.9.1751 11006345 PMC149083

[B51] Schultz C. J. Rumsewicz M. P. Johnson K. L. Jones B. J. Gaspar Y. M. Bacic A. (2002). Using genomic resources to guide research directions. The arabinogalactan protein gene family as a test case. Plant Physiol. 129, 1448–1463. 10.1104/pp.003459 12177459 PMC166734

[B52] Seifert G. J. Roberts K. (2007). The biology of arabinogalactan proteins. Annu. Rev. Plant. Biol. 58, 137–161. 10.1146/annurev.arplant.58.032806.103801 17201686

[B53] Serpe M. D. Nothnagel E. A. (1994). Effects of Yariv phenylglycosides on rosa cell-suspensions-evidence for the involvement of arabinogalactan-proteins in cell proliferation. Planta 193, 542–550. 10.1007/BF02411560

[B54] Shi H. Kim Y. Guo Y. Stevenson B. Zhu J. K. (2003). The arabidopsis SOS5 locus encodes a putative cell surface adhesion protein and is required for normal cell expansion. Plant Cell 15, 19–32. 10.1105/tpc.007872 12509519 PMC143448

[B55] Shimizu M. Igasaki T. Yamada M. Yuasa K. Hasegawa J. Kato T.. (2005). Experimental determination of proline hydroxylation and hydroxyproline arabinogalactosylation motifs in secretory proteins. Plant J. 42, 877–889. 10.1111/j.1365-313X.2005.02419.x 15941400

[B56] Showalter A. M. (1993). Structure and function of plant-cell wall proteins. Plant Cell 5, 9–23. 10.1105/tpc.5.1.9 8439747 PMC160246

[B57] Showalter A. M. (2001). Arabinogalactan-proteins: structure, expression and function. Cell Mol. Life Sci. 58, 1399–1417. 10.1007/PL00000784 11693522 PMC11337269

[B58] Showalter A. M. Basu D. (2016). Extensin and arabinogalactan-protein biosynthesis: glycosyltransferases, research challenges, and biosensors. Front. Plant Sci. 7:814. 10.3389/fpls.2016.00814 27379116 PMC4908140

[B59] Showalter A. M. Keppler B. D. Liu X. Lichtenberg J. Welch L. R. (2016). Bioinformatic identification and analysis of hydroxyproline-rich glycoproteins in *Populus trichocarpa*. BMC Plant Biol. 16:229. 10.1186/s12870-016-0912-3 27769192 PMC5073881

[B60] Showalter A. M. Keppler B. Lichtenberg J. Gu D. Welch L. R. (2010). A bioinformatics approach to the identification, classification, and analysis of hydroxyproline-rich glycoproteins. Plant Physiol. 153, 485–513. 10.1104/pp.110.156554 20395450 PMC2879790

[B61] Shpak E. Barbar E. Leykam J. F. Kieliszewski M. J. (2001). Contiguous hydroxyproline residues direct hydroxyproline arabinosylation in *Nicotiana tabacum*. J. Biol. Chem. 276, 11272–11278. 10.1074/jbc.M011323200 11154705

[B62] Shpak E. Leykam J. F. Kieliszewski M. J. (1999). Synthetic genes for glycoprotein design and the elucidation of hydroxyproline-O-glycosylation codes. Proc. Natl. Acad. Sci. U.S.A. 96, 14736–14741. 10.1073/pnas.96.26.14736 10611282 PMC24717

[B63] Silva N. F. Goring D. R. (2002). The proline-rich, extensin-like receptor kinase-1 (PERK1) gene is rapidly induced by wounding. Plant Mol. Biol. 50, 667–685. 10.1023/A:1019951120788 12374299

[B64] Sun W. Xu J. Yang J. Kieliszewski M. J. Showalter A. M. (2005). The lysine-rich arabinogalactan-protein subfamily in Arabidopsis: gene expression, glycoprotein purification and biochemical characterization. Plant Cell Physiol. 46, 975–984. 10.1093/pcp/pci106 15840645

[B65] Tan L. Eberhard S. Pattathil S. Warder C. Glushka J. Yuan C.. (2013). An Arabidopsis cell wall proteoglycan consists of pectin and arabinoxylan covalently linked to an arabinogalactan protein. Plant Cell 25, 270–287. 10.1105/tpc.112.107334 23371948 PMC3584541

[B66] van Hengel A. J. Roberts K. (2003). AtAGP30, an arabinogalactan-protein in the cell walls of the primary root, plays a role in root regeneration and seed germination. Plant J. 36, 256–270. 10.1046/j.1365-313X.2003.01874.x 14535889

[B67] Vorwerk S. Somerville S. Somerville C. (2004). The role of plant cell wall polysaccharide composition in disease resistance. Trends Plant Sci. 9, 203–209. 10.1016/j.tplants.2004.02.005 15063871

[B68] Wu H. M. Wang H. Cheung A. Y. (1995). A pollen tube growth stimulatory glycoprotein is deglycosylated by pollen tubes and displays a glycosylation gradient in the flower. Cell 82, 395–403. 10.1016/0092-8674(95)90428-X 7634329

[B69] Zang L. Zheng T. Chu Y. Ding C. Zhang W. Huang Q.. (2015). Genome-wide analysis of the fasciclin-like arabinogalactan protein gene family reveals differential expression patterns, localization, and salt stress response in Populus. Front. Plant Sci. 6:1140. 10.3389/fpls.2015.01140 26779187 PMC4688393

[B70] Zhang Y. Yang J. Showalter A. M. (2011). AtAGP18, a lysine-rich arabinogalactan protein in *Arabidopsis thaliana*, functions in plant growth and development as a putative co-receptor for signal transduction. Plant Signal. Behav. 6, 855–857. 10.4161/psb.6.6.15204 21849816 PMC3218486

