# Supplementary material for: Bioinformatics Prediction and Evolution Analysis of Arabinogalactan Proteins in the Plant Kingdom
Source: Front Plant Sci. 2017 Jan 26;8:66. doi: 10.3389/fpls.2017.00066 (PMC5266747; doi:10.3389/fpls.2017.00066)
Supplement: Supplementary file 1 [file Table1.DOC]

| **Supplementary Table S1.** **Sequence collections of 325 already-known AGPs from 22 plant species** | | | | | | | | | |
| --- | --- | --- | --- | --- | --- | --- | --- | --- | --- |
| **Species** | **C^a^** | **KC** | **Pep** | **FLA** | **PAG** | **XYLP** | **NC** | **HAE** | **Total** |
| *Arabidopsis thaliana* | 22 | 3 | 16 | 21 | 30 | 13 | 6 | 4 | 115 |
| *Oryza sativa* | 11 | 2 | 15 | 27 | 38 | 21 | 3 | - | 117 |
| *Solanum lycopersicum* | 2 | 1 | 2 | 21 | 3 | - | 2 | - | 31 |
| *Gossypium hirsutum* | 1 | -^b^ | - | 23 | 1 | - | 1 | - | 26 |
| *Nicotiana tabacum* | - | - | - | - | 1 | - | 2 | - | 3 |
| *Nicotiana alata* | 1 | 1 | - | - | - | - | 2 | 1 | 8 |
| *Malus domestica* | - | - | 3 | - | - | - | - | - | 3 |
| *Hordeum vulgare* | - | - | - | - | - | - | 1 | - | 1 |
| *Brachypodium distachyon* | - | - | - | - | - | - | 1 | - | 1 |
| *Brassica campestris* | 1 | - | - | - | - | - | - | - | 1 |
| *Brassica napus* | 2 | - | - | - | - | - | - | - | 2 |
| *Brassica rapa* | - | - | 1 | - | - | - | - | - | 1 |
| *Cucumis sativus* | - | 1 | - | - | - | - | - | - | 1 |
| *Daucus carota* | - | - | - | - | - | - | 1 | - | 1 |
| *Eucalyptus nitens* | - | - | - | 3 | - | - | - | - | 3 |
| *Petunia hybrida* | - | - | - | - | - | - | 1 | - | 1 |
| *Physcomitrella patens* | 2 | - | 3 | 2 | - | - | - | - | 7 |
| *Pinus taeda* | - | 1 | - | - | - | - | - | - | 1 |
| *Triticum aestivum* | - | - | - | - | - | - | 1 | - | 1 |
| *Vicia faba* | - | - | - | - | 1 | - | - | - | 1 |
| *Zea mays* | - | - | - | - | - | - | 1 | - | 1 |
| *Zinnia elegans* | - | - | - | 1 | - | 1 | - | - | 2 |

^a^ C, classical AGPs; KC, Lys-rich classical AGPs; Pep, AG-peptides; FLA, fasciclin-like AGPs; PAG, phytocyanin-like AGPs; XYLP, xylogen-like AGPs; NC, non-classical AGPs; HAE, AGP-Extensin hybrid. ^b^ “-” means “not available”.
