# Supplementary material for: Bioinformatics Prediction and Evolution Analysis of Arabinogalactan Proteins in the Plant Kingdom
Source: Front Plant Sci. 2017 Jan 26;8:66. doi: 10.3389/fpls.2017.00066 (PMC5266747; doi:10.3389/fpls.2017.00066)
Supplement: Supplementary file 3 [file Table3.DOC]

| **Supplementary Table S3. Compositions of twenty amino acids in** **325 already-known AGPs** | | | | | |
| --- | --- | --- | --- | --- | --- |
| **Amino acid^a^**  **(three letter)** | **Amino acid**  **(one letter)** | **Total number^b^** | **Frequency of given amino acid in 325 sequences** | **Average number of given amino acid per sequence^c^** | **Percentage of given amino acid in all amino acids (%)^d^** |
| Ala | A | 9703 | 325 | 29.86 | 13.09 |
| Ser | S | 7978 | 325 | 24.55 | 10.76 |
| Pro | P | 7762 | 325 | 23.88 | 10.47 |
| Leu | L | 6341 | 325 | 19.51 | 8.55 |
| Val | V | 5565 | 325 | 17.12 | 7.51 |
| Thr | T | 5261 | 324 | 16.24 | 7.10 |
| Gly | G | 5007 | 325 | 15.41 | 6.75 |
| Lys | K | 3182 | 309 | 10.30 | 4.29 |
| Asp | D | 2949 | 307 | 9.61 | 3.98 |
| Asn | N | 2775 | 294 | 9.44 | 3.74 |
| Phe | F | 2762 | 316 | 8.74 | 3.72 |
| Ile | I | 2647 | 320 | 8.27 | 3.57 |
| Glu | E | 2215 | 304 | 7.29 | 2.99 |
| Gln | Q | 1899 | 305 | 6.23 | 2.56 |
| Arg | R | 1844 | 292 | 6.32 | 2.49 |
| Tyr | Y | 1842 | 291 | 6.33 | 2.48 |
| Met | M | 1572 | 287 | 5.48 | 2.12 |
| His | H | 1559 | 325 | 4.80 | 2.10 |
| Cys | C | 952 | 266 | 3.58 | 1.28 |
| Trp | W | 385 | 188 | 2.05 | 0.52 |
| ^a^ The order of twenty amino acids was displayed according to their total number from high to low. ^b^ The sum of given amino acid in all 325 sequences. ^c^ The value was calculated by using the total number of given amino acid divide the number of sequences with given amino acid. ^d^ The value was calculated by using total number of given amino acid divide total number of amino acids of 325 already-known AGPs. | | | | | |
