# Supplementary material for: Bioinformatics Prediction and Evolution Analysis of Arabinogalactan Proteins in the Plant Kingdom
Source: Front Plant Sci. 2017 Jan 26;8:66. doi: 10.3389/fpls.2017.00066 (PMC5266747; doi:10.3389/fpls.2017.00066)
Supplement: Supplementary file 4 [file Table4.DOC]

| **Supplementary Table S4.** **Glycomodule counts of Pro-Ala, Pro-Ser, and Pro-Thr in 87 AGP-like sequences** | | | | | |
| --- | --- | --- | --- | --- | --- |
| **Glycomodule** | **Number** | **Glycomodule** | **Number** | **Glycomodule** | **Number** |
| GPA | 22 | GPS | 20 | GPT | 7 |
| VPA | 17 | VPS | 1 | VPT | 4 |
| LPA | 3 | LPS | 8 | LPT | 1 |
| IPA | 1 | IPS | 5 | IPT | 1 |
| FPA | 1 | FPS | 1 | FPT | 1 |
| YPA | 0 | YPS | 1 | YPT | 0 |
| WPA | 2 | WPS | 0 | WPT | 0 |
| CPA | 0 | CPS | 1 | CPT | 0 |
| MPA | 0 | MPS | 2 | MPT | 2 |
| NPA | 0 | NPS | 2 | NPT | 0 |
| QPA | 1 | QPS | 0 | QPT | 1 |
| DPA | 1 | DPS | 0 | DPT | 0 |
| EPA | 2 | EPS | 1 | EPT | 6 |
| KPA | 3 | KPS | 4 | KPT | 1 |
| RPA | 2 | RPS | 0 | RPT | 1 |
| HPA | 0 | HPS | 0 | HPT | 0 |
| Total | 55 | Total | 46 | Total | 25 |
