# Supplementary material for: Bioinformatics Prediction and Evolution Analysis of Arabinogalactan Proteins in the Plant Kingdom
Source: Front Plant Sci. 2017 Jan 26;8:66. doi: 10.3389/fpls.2017.00066 (PMC5266747; doi:10.3389/fpls.2017.00066)
Supplement: Supplementary file 8 [file Table8.DOC]

| **Supplementary Table S8. Number of proteins in chimeric subfamilies** | | | | | | | | | |
| --- | --- | --- | --- | --- | --- | --- | --- | --- | --- |
| **Species** | **PK^a^** | **FH2** | **GH** | **POeI** | **LRR** | **X8** | **PMEI** | **PCL** | **SGNH** |
| *Actinidia chinensis* | 6 | 1 | 2 | -^b^ | - | 1 | 1 | - | - |
| *Amborella trichopoda* | - | - | - | - | - | 1 | 1 | - | - |
| *Arabidopsis lyrata* | 8 | 3 | 1 | 2 | 1 | - | 1 | - | 1 |
| *Arabidopsis thaliana* | 7 | 4 | 2 | 2 | 2 | - | 2 | 1 | - |
| *Beta vulgaris* | 6 | 2 | 2 | 1 | 2 | 1 | - | - | - |
| *Brachypodium distachyon* | 23 | 6 | 2 | 4 | 2 | 1 | 3 | 1 | 3 |
| *Brassica oleracea* | 11 | 5 | 1 | 5 | - | - | 3 | 1 | - |
| *Brassica rapa* | 13 | 11 | 2 | 5 | 5 | 2 | 2 | - | - |
| *Cajanus cajan* | 4 | 3 | - | 1 | - | - | 1 | 2 | - |
| *Capsella rubella* | 11 | 6 | 2 | 3 | 2 | - | 1 | - | - |
| *Capsicum annuum* | 5 | 3 | 1 | 3 | 1 | - | 1 | 1 | - |
| *Carica papaya* | 1 | 1 | 2 | 1 | - | 1 | 2 | 2 | - |
| *Chlamydomonas reinhardtii* | 1 | - | 1 | - | - | - | - | - | - |
| *Cicer arietinum* | 4 | 2 | 1 | 1 | 1 | 1 | 1 | - | 1 |
| *Citrullus lanatus* | 6 | 2 | 2 | 2 | 1 | 2 | 1 | - | - |
| *Citrus sinensis* | 7 | 4 | 1 | 1 | 3 | 2 | 1 | 1 | 1 |
| *Cucumis sativus* | 5 | 2 | 1 | 2 | - | 3 | - | - | 1 |
| *Elaeis guineensis* | 4 | 7 | 2 | 1 | 2 | 3 | 1 | - | - |
| *Eucalyptus grandis* | 12 | 4 | 1 | 2 | 3 | 3 | 1 | 2 | 4 |
| *Fragaria vesca* | 8 | 3 | 2 | 1 | - | 1 | - | 2 | 1 |
| *Glycine max* | 27 | 14 | 2 | 2 | 1 | 6 | 1 | 7 | 3 |
| *Gossypium raimondii* | 7 | 4 | 3 | 2 | 2 | 2 | 1 | 1 | 1 |
| *Hordeum vulgare* | 7 | 2 | 1 | 4 | 3 | 4 | 1 | 3 | 2 |
| *Lotus japonicus* | 9 | 5 | 2 | 1 | 2 | 3 | - | 3 | 2 |
| *Malus domestica* | 10 | 5 | 4 | 1 | 1 | 2 | - | 2 | 1 |
| *Medicago truncatula* | 11 | 5 | 1 | 11 | 1 | 5 | - | 4 | 1 |
| *Musa acuminata* | 9 | 1 | - | 1 | 1 | 1 | - | 1 | - |
| *Nelumbo nucifera* | 8 | 5 | 3 | 1 | 1 | - | - | - | - |
| *Oryza sativa* | 33 | 10 | 5 | 6 | 4 | 3 | 3 | 2 | 3 |
| *Phalaenopsis equestris* | 5 | 4 | - | - | - | - | - | - | - |
| *Phaseolus vulgaris* | 17 | 6 | - | 3 | - | 1 | - | 1 | 1 |
| *Physcomitrella patens* | 8 | - | 2 | - | 1 | - | - | - | - |
| *Picea abies* | 4 | 1 | 2 | 1 | 2 | 1 | - | - | - |
| *Populus trichocarpa* | 15 | 5 | 2 | 2 | 1 | 5 | 4 | 4 | 1 |
| *Prunus mume* | 7 | 4 | 1 | 2 | - | 1 | 1 | 2 | 2 |
| *Prunus persica* | 5 | 3 | 1 | - | - | - | 1 | 1 | - |
| *Pyrus bretschneideri* | 12 | 9 | 6 | 3 | 2 | - | 1 | 3 | 2 |
| *Ricinus communis* | 3 | 2 | 1 | 1 | 2 | - | 3 | 1 | 1 |
| *Selaginella moellendorffii* | 2 | - | 2 | 3 | 1 | - | - | - | 1 |
| *Solanum lycopersicum* | 9 | 6 | 2 | 2 | 1 | - | - | 1 | 2 |
| *Solanum tuberosum* | 5 | 6 | 1 | 2 | 2 | 1 | 2 |  | 2 |
| *Sorghum bicolor* | 35 | 7 | 2 | 4 | 3 | 4 | 4 | 3 | 5 |
| *Theobroma cacao* | 10 | 6 | 2 | 1 | 1 | 2 | 2 |  | 1 |
| *Triticum urartu* | 9 | 3 | 2 | 2 | 1 | 5 | 2 | 2 | 1 |
| *Utricularia gibba* | 5 | 2 | - | - | 2 | 1 | - | 1 | - |
| *Vitis vinifera* | 12 | 4 | 4 | 1 | 1 | 2 | - | - | - |
| *Zea mays* | 23 | 10 | 1 | 5 | 1 | 4 | 2 | 6 | 5 |
| Total number | 449 | 198 | 80 | 98 | 62 | 75 | 51 | 61 | 49 |
| ^a^ PK, protein kinase-like AGPs; FH2, Formin homology 2-like AGPs; GH, Glycosyl hydrolase-like AGPs; POeI, Pollen Ole e I-like AGPs; LRR, Leucine-rich repeats-like AGPs; X8, X8-like AGPs; PMEI, Pectin methylesterase inhibitor-like AGPs; PCL, Pectate lyase-like AGPs; SGNH, SGNH hydrolase-like AGPs. | | | | | | | | | |
