# Supplementary material for: Bioinformatics Prediction and Evolution Analysis of Arabinogalactan Proteins in the Plant Kingdom
Source: Front Plant Sci. 2017 Jan 26;8:66. doi: 10.3389/fpls.2017.00066 (PMC5266747; doi:10.3389/fpls.2017.00066)
Supplement: Supplementary file 11 [file Image1.PDF]

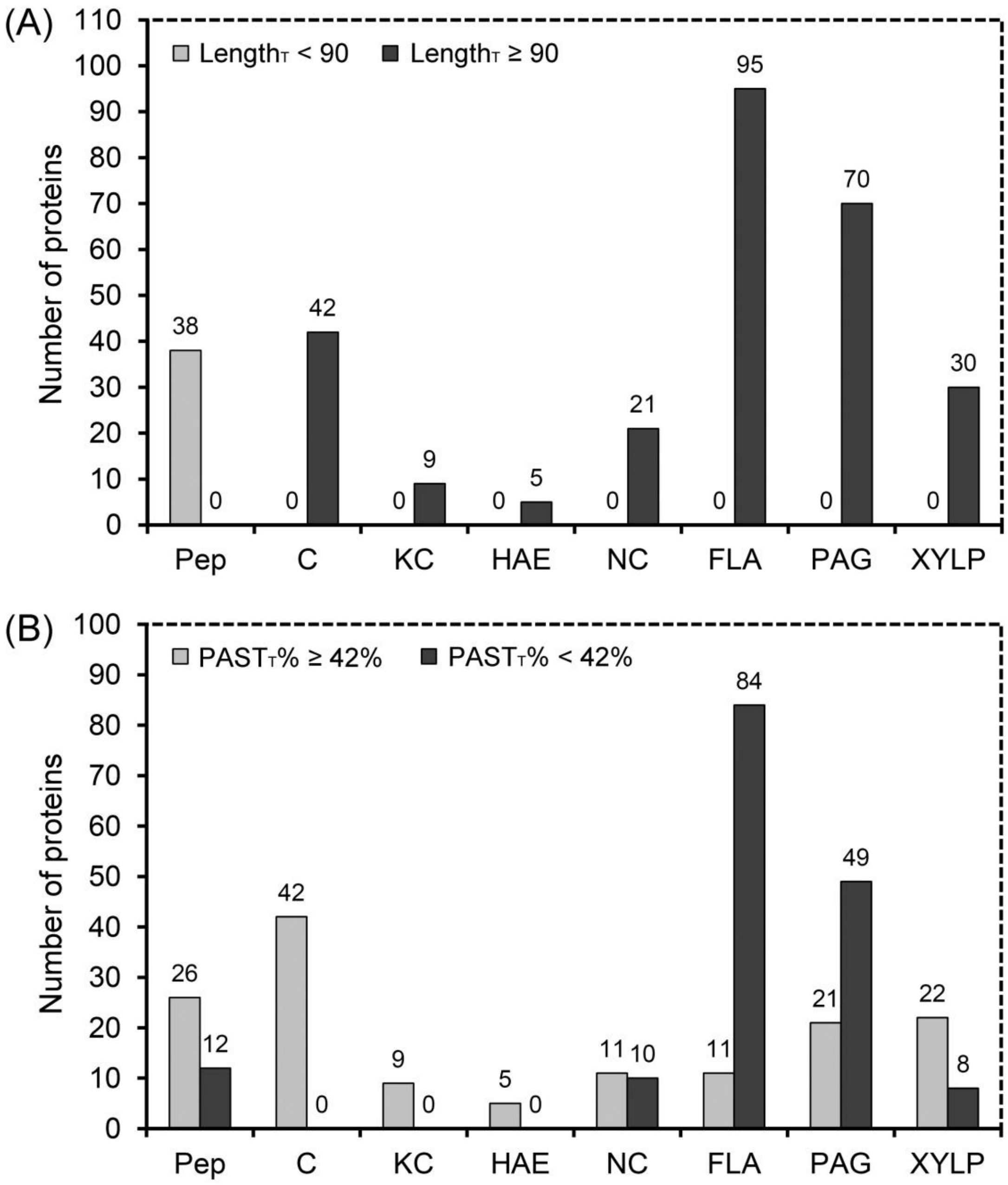


**Supplementary Figure S1. The thresholds to distinguish AGPs into three groups.** Number of proteins is detected under the thresholds of 42% $PAST_T$% (A) and 90 LengthT (B). C, Classical AGPs; KC, Lys-rich classical AGPs; HAE, AGP-Extensin hybrid; Pep, AG-peptides; Chimeric, Chimeric AGPs including fasciclin-like AGPs, phytocyanin-like AGPs, and xylogen-like AGPs; NC, non-classical AGPs.
