# Supplementary material for: Bioinformatics Prediction and Evolution Analysis of Arabinogalactan Proteins in the Plant Kingdom
Source: Front Plant Sci. 2017 Jan 26;8:66. doi: 10.3389/fpls.2017.00066 (PMC5266747; doi:10.3389/fpls.2017.00066)
Supplement: Supplementary file 12 [file Image2.PDF]

LOC_Os07g49240.1 [Chimeric, Protein kinase] PAST_{T}% = 33.33% GlycoIndex = 0.25 PAST_{P}% = 75.00%

MALPLLRLLLLLVLSSVPLSCQLAHSTAADTQNWGQTSVVHLRNAHTRKLLGLLDDISGRTGSLHALLLEESPKQAPPH
HHNRHGGHHRAAHTPAPSPAPSPSPFTAPPKSASPAAITIPISPSTPQPKAESNPAVEDAPAQPRHSWRNYGLVTAG
SAVFLVMTIASVIYCRAKKVGTVRPWATGLSGQLQRAFVTGVPSLKRSELEAACEDFSNIIGSTSSCMLYKGTLSSGVEI
AVLTSSTESGKEWSKECESQYRKKITNLSKVSHKNFMNLLGYCEEENLFTRAMVFEYAPNGTLFEYLHVREAENLDW
MARVRISMGIAYCLEHMHQLNPPVVPRNFNSTTIYLTDDFAAKVSDLDFWNDSKGSFNSATSDETVMVEIDSMVHQYG
IILLEILTGRVPYSESDGPLEHWASGYFEGKMTLAELIDPSLGSFPEDAARALCDVARWCIEPEPSKRPLMSQVAGRMK
EITSLGPEGATPKVSPLWWAELEIMSGQAT

LOC_Os02g50570.1 [**Chimeric, Formin homology 2] PAST_{T}% = 40.68% GlycoIndex = 0.25 PAST_{P}% = 85.22%**

MAPAPSPTPLPLFLLLLLLVGVAPLAAAQGQNIQTRFPSTRTPAFATPPPITSPSPSPGTPTATPSSSPPSSSGKRSDIA
VAVVSTALSSFAVSGLAFFLFLRHGKKRELTEAGGAGQHYGGAQGGALTGKRPEREPKRPARGNMVDENGLDAIYWR
EFEKEGDGGRGRKPPASRRPPQPPPPRPYRAERRQDAHESSAPSPPRSRKNRIDQEPLIPRGSLDSASAEFDESLYA
PSAGSTSSFSVAAAEAYARPPSTPAITAVSSVPRSSPSPAPAPAARPASPSPSLPLPPGRESPSRPQSIAAAAVASPAP
PPPPPPKPAAAAPPPPPPPKAAPPPPPPKGPPPPPPAKGPPPPPPPPKGPSPPPPPPPPGGKKGGPPPPPPKGGASRP
PAAPGVPTGSADQQAKLKPLHWDKVNVAATDHSMVWDNITGGSFNLDEGIIEALFGTAAVNRKTKPADSKDASGGST
SAGLGRSNSPEQIFLLEPRKSHNISIILRSLTVGREEIIDALLNGHTELSTEVLEKLSRLNISKEEENTLLKFSGNPDRLAP
AEFFLLRLLLDVPSPFARVNALLFKANYAAEVAQLKQSLRTLEMASQELRTKGLFFKLLEAVLKAGNRMNAGTARGNA
QAFNLTALRKLSDVKSTDGSTTLLHFVIEEVVRSEGKRLAINRNYSLRRSGSLAKSTDGGNPAASSTSQGPSREERQN
EYLNLGLPIVGGLSTEFANVKKAALVDYDTVVNECAILGNRLAGTKKLLETYGDDGFARGLRGFVKAAEQELNELKGN
QEKVLELVQRTTEYYHTGATKDKNAHPLQLFIIVRDFLGMVDQACVDIKRKLQQQKKPTPPPSSSQPAAPAATTKGAA
DDAPAPAQKPPEEVDSKRKRVMPRFPNLPAHFMKDNADSDSSSDEE

LOC_Os01g44970.1 [Chimeric, Pectate lyase] PAST_{T}% = 29.31% GlycoIndex = 0.23 PAST_{P}% = 62.32%

MKLRVKGLGLLLLLVLLALCSTIDVCDARRGKHWRPRSSPSSSLLRNKGKGKKGSSNRQHGSNRPSPKPPLSPPPSP
GNGKGYQSPYQPSPSPSPNAPVSPSPVNGSGHASPKSPTPSCGKGNQPPSRPTPTSPQGAVFNVVDFGAKGDGVS
DDTKAFEAAWAAACKQGASTVVVPSELEFLVGPISFSGPYCKPNILFQLDGTIVAPTSAKAWGSGLLQWIEFTKLNGVS
IQGNGIINGRGQQWWTYSDIDDDEDDDTQYDVEFERMPQVKPTALRFYGSFNVVVAGITIVNSSQCHLKFDSCQGVM
VHDVTISSPENSLNTDGIHLQNSKDVSIHHTNLACGDDCVSIQTGCSNINIHNVNCGPGHGISIGGLGRDNTKACVSNV
TVRDVNMFRTMTGVRIKTWQGGLGLVQDVRFSNIQVSEVQTPIIIDQFYCDKRTCSNQTSAVAVSGVQYENIRGTFTIK
PVHFACSDSSPCSGITLTGVQLRPVQIPHYRLNDPFCWQAFGELYTPTVPPIACLHLGKPAGNNLQSYHDLC

LOC_Os07g14340.1 [Chimeric, Pectin methylesterase inhibitor] PAST_{T}% = 50.14% GlycoIndex = 0.22 PAST_{P}% = 74.38%

MAMARSQLAAVFLAVLVSVAPLAGAADNLQDACNRTLFPKVCIQALTTNPESRTANARRLAELSVYVAAEVGTAVAAFA
HHELNGVKEDALFKCVDSCSDDIEEAVAHLSALTRELTDAKFLEVKAWLSATLGGSSTCEDTCKDAPISEIKNAVVTKSL
EFEKLLRVTLDLITEASGSMSADVALPPATGGGGGGYGYESSSAAAAPAPSESDSDVGSGSGSAASAPGPSPSDDTG
YGGSSGSPSSSPSSSPSSSPSGSPSSSPSGSPSGSPAGSPAGGPTAGGPASGPSSYGAASGPAEGPSSSGAASGP
AEGPSPSGAAGPAEGPSSYGSTEGPSPSPSSSGSADAPGPGASAPDSEEY

Supplementary Figure S2. Sequence characteristics of representative chimeric AGPs in *Oryza sativa.* Four AGP sequences homologous to Protein kinase, Formin homology 2, Pectate lyase, and Pectin methylesterase inhibitor are selected to show the constitution of different conversed domains and AGP-like sequences. Red colored sequences indicate putative AG glycomodules.
